# Integrated multi-omics profiling reveals the role of the DNA methylation landscape in shaping biological heterogeneity and clinical behaviour of metastatic melanoma

**DOI:** 10.1186/s13046-025-03474-9

**Published:** 2025-07-18

**Authors:** Andrea Anichini, Francesca P. Caruso, Vincenzo Lagano, Teresa M. R. Noviello, Rossella Tufano, Gabriella Nicolini, Alessandra Molla, Ilaria Bersani, Francesco Sgambelluri, Alessia Covre, Maria F. Lofiego, Sandra Coral, Anna Maria Di Giacomo, Elena Simonetti, Barbara Valeri, Mara Cossa, Filippo Ugolini, Sara Simi, Daniela Massi, Massimo Milione, Andrea Maurichi, Roberto Patuzzo, Mario Santinami, Michele Maio, Michele Ceccarelli, Roberta Mortarini, On behalf of the EPigenetic Immune-oncology Consortium Airc (EPICA) investigators

**Affiliations:** 1 Department of Experimental Oncology, Fondazione IRCCS Istituto Nazionale Dei Tumori, Milan, Italy; 2 Biogem Institute of Molecular Biology and Genetics, Ariano Irpino, Italy; 3 Department of Electrical Engineering and Information Technologies, University of Naples Federico II, Naples, Italy; 4 Sylvester Comprehensive Cancer Center, and Department of Public Health Sciences, Miller School of Medicine, University of Miami, Miami, FL USA; 5 University of Siena, Italy, and Center for Immuno-Oncology, Department of Oncology, University Hospital of Siena, Siena, Italy; 6 NIBIT Foundation Onlus, Siena, Italy; 7 Department of Advanced Diagnostics, Fondazione IRCCS Istituto Nazionale Dei Tumori, Milan, Italy; 8 Section of Anatomic Pathology, Department of Health Sciences, University of Florence, Florence, Italy; 9 Department of Oral and Maxillofacial Surgery, New York University - College of Dentistry, New York, USA; 10 Department of Surgical Oncology, Fondazione IRCCS Istituto Nazionale Dei Tumori, Milan, Italy

**Keywords:** Melanoma, DNA methylation, Immune contexture, Immune checkpoint blockade, DNMT inhibitor

## Abstract

**Background:**

We developed an integrated multi-omics analysis in metastatic melanoma (MM) cohorts to associate DNA methylation profiles with tumor progression, survival, response to adjuvant immunotherapy, structure of the tumor immune microenvironment and transcriptional programs of immunity and melanoma differentiation.

**Methods:**

Lesions (*n* = 191) from a fully annotated, retrospective cohort of 165 AJCC 8th Stage III and IV melanoma patients (EPICA cohort) were characterized by reduced representation bisulfite sequencing, RNA sequencing, whole exome sequencing, quantitative immunohistochemistry and multiplex immunofluorescence analysis. The TCGA melanoma datasets were used for validation. Pre-therapy lesions (*n* = 28) from a cohort of MM patients treated with adjuvant immune checkpoint blockade were characterized for the DNA methylation profile. Impact of a DNMT inhibitor on DNA methylation and transcriptomic profiles of melanoma cell lines was investigated by EPIC arrays and Clariom S arrays.

**Results:**

Four tumor subsets (i.e. DEMethylated, LOW, INTermediate and CIMP) with progressively increasing levels of DNA methylation were identified in EPICA, TCGA MM and TCGA primary melanoma cohorts. EPICA patients with LOW methylation tumors exhibited a significantly longer survival and a lower progression rate to more advanced AJCC stages, compared to patients with CIMP tumors. In an adjuvant immune checkpoint blockade cohort, patients with DEM/LOW pre-therapy lesions showed significantly longer relapse-free survival compared to those with INT/CIMP lesions. RNA-seq data analysis revealed that LOW and CIMP EPICA tumors showed opposite activation of master molecules influencing prognostic target genes, and differential expression of immunotherapy response and melanoma differentiation signatures. Compared to CIMP tumors, LOW lesions showed enrichment for CD8^+^ TCF-1^+^ PD-1^+^ TIM-3^−^ pre-exhausted and CD8^+^ TCF-1^−^ PD-1^+^ TIM-3^+^ exhausted T cells, more frequent retention of HLA Class I antigens and a de-differentiated melanoma phenotype. The differentiation and immune-related transcriptional features associated with LOW vs CIMP lesions were tumor-intrinsic programs retained in-vitro by melanoma cell lines. Consistently, treatment of differentiated melanoma cell lines with a DNMT inhibitor induced global DNA de-methylation, promoted de-differentiation and upregulated viral mimicry and IFNG predictive signatures of immunotherapy response.

**Conclusions:**

These results reveal the biological, prognostic and therapeutic relevance of DNA methylation classes in MM and support methylome targeting strategies for precision immunotherapy.

**Supplementary Information:**

The online version contains supplementary material available at 10.1186/s13046-025-03474-9.

## Background

Nonmutational epigenetic reprogramming, one of the enabling characteristics contributing to the acquisition of the hallmarks of cancer [1], refers to the plethora of epigenetic mechanisms affecting gene expression independently from genome instability and gene mutation. Among such mechanisms, altered DNA methylation at CpG dinucleotides contributes to tumorigenesis and to cancer evolution [2]. Early studies identified a progressive reduction in the overall 5-methylcytosine content along with the transition from benign lesions to primary malignant tumors and then to metastatic lesions [3]. Conversely, cancer-specific DNA hypermethylation at CpG islands, associated with transcriptional silencing of tumor suppressors, was subsequently discovered [4]. In cutaneous melanoma, both global and gene-specific DNA methylation changes play a role in melanocyte transformation [5], contribute to shape the melanoma differentiation phenotype [6, 7] and impact on tumor progression [8–10] and clinical outcome [8, 11–15].

In primary melanomas cohorts (*n* = 47 and *n* = 89) Thomas et al. [16] and Conway et al. [14] found three methylation classes (Low, Intermediate, High/CIMP). The CIMP class was associated with Breslow thickness in one of these studies [16] and with higher AJCC Stage, age at diagnosis ≥ 65 years, lower tumor-infiltrating lymphocyte grade and worse melanoma-specific survival in the other study [14]. A two-fold higher likelihood of 5-year death for patients with primary melanomas in the CIMP or intermediate methylation classes, compared to the Low methylation class, has been reported in a cohort of 422 primary melanoma patients [17]. In a metastatic melanoma (MM) cohort (*n* = 50), two groups with the highest and the lowest global methylation levels showed differential expression of immune-related and proliferative signatures [15]. In a cohort of *n* = 332 primary and MM lesions from previously untreated patients the Cancer Genome ATLAS (TCGA) network [18] identified four methylation subsets, with increasing global methylation levels (hypomethylated, normal-like, hypermethylated and CIMP). The normal-like subset was associated with a longer survival compared to the CIMP class, although patients were not stratified for tumor stage [18].

Despite these initial intriguing insights, the overall biological significance and potential therapeutic relevance of methylation-defined subsets in melanoma remain underexplored, as several key gaps in knowledge still need to be addressed. First, the impact of DNA methylation classes on the clinical outcome of patients, and on disease progression to more advanced stages, has poorly understood molecular underpinnings. Second, the potential association of the methylation subsets with transcriptional programs of response to immune checkpoint blockade (ICB) therapies and with the structure of the immune contexture remain to be fully deciphered. Third, the association of DNA methylation classes with response to ICB therapies remains to be clarified. Fourth, the direct role of DNA methylation in shaping melanoma transcriptional programs of immunity and differentiation needs to be verified by cause-effect experiments. Fifth, the development of more effective immunotherapeutic approaches is urgently needed for advanced melanoma, therefore it is crucial to assess whether DNA methylation of melanoma is a suitable target for immune reprogramming of tumors. To address these issues, we carried out an integrative analysis of multi-omics data generated in tumor lesions from a fully annotated cohort of Stage III/IV patients (EPICA). Among the four methylation-defined subsets that were identified, the LOW and CIMP lesions expressed opposite melanoma differentiation and immune-related programs, distinct tumor immune contextures and divergent clinical evolution. Results from an independent adjuvant ICB MM cohort supported the clinical relevance of the DNA methylation classification. Moreover, global DNA demethylation, induced by a DNMT inhibitor (DNMTi), reprogrammed MITF^HI^/PMEL^HI^ melanoma cells towards a de-differentiated profile associated with upregulation of predictive signatures of immunotherapy response.

## Materials and methods

### Melanoma patients and neoplastic tissues

Tumor samples (*n* = 191) from the EPICA cohort of MM lesions were obtained based on informed consent from *n* = 165 Stage III and IV patients according to AJCC 8th edition. The study was conducted according to the Declaration of Helsinki Principles and following approval by the Ethics Committee of Fondazione IRCCS Istituto Nazionale dei Tumori, Milan, Italy (protocol number INT 170/18). Patient and lesion eligibility criteria are described in Supplemental Methods. Relevant demographic and clinicopathological data of the EPICA cohort are listed in patient centric form in Supplemental Table S1A and in lesion centric form in Supplemental Table S1B. Tumor samples (*n* = 28) from an additional cohort of MM patients (Supplemental Table S2) were obtained from *n* = 28 Stage III or IV-resected (according to AJCC 8th edition) patients treated with adjuvant ICB therapy in daily practice or within clinical trials (CA-209–238 (NCT02388906) [19]; CA-209–915 (NCT03068455) [20] at the Center for Immuno-Oncology, Department of Oncology, University Hospital of Siena, Siena, Italy. All patients provided an informed consent.

### DNA extraction from FFPE sections, quality controls and reduced representation bisulfite sequencing (RRBS)

Isolation of total DNA from FFPE sections was performed by using Maxwell (R) RSC FFPE Plus DNA Kit (Promega, Madison, WI, USA) according to the manufacturer’s protocol. Quality controls (QCs) were performed on the TapeStation 4200 system using the Genomic DNA High Sensitivity ScreenTape Assay (Agilent Technologies, Santa Clara, CA, USA). All samples had a concentration range between 5–10 ng/µl and a DNA integrity number (DIN) between 2 and 5. Library preparation protocols were adapted to optimize the result obtained with low integrity samples. Isolated DNA (300 ng) was exploited to perform further methylation analyses. Briefly, Ovation® RRBS Methyl-Seq (Tecan/NuGEN, Redwood City, CA) was used for library preparation following the manufacturer’s instructions. Unmethylated lambda phage DNA was spiked-in to estimate the bisulphite conversion rate. DNA samples were quantified with Qubit 2.0 Fluorometer (Invitrogen, Carlsbad, CA). Final libraries were checked with both Qubit 2.0 Fluorometer (Invitrogen) and Bioanalyzer HS DNA assay (Agilent Technologies). Libraries were then prepared for sequencing and sequenced on paired-end 150 bp mode on NovaSeq6000 (Illumina, San Diego, CA).

### Whole-exome sequencing (WES)

WES was performed using Human Comprehensive Exome kit (Twist Bioscience, San Francisco, CA, USA) starting from 200 ng total input. Samples with DIN < 3 were fragmented for 4 min at 32 °C to maintain 200 bp. Half-volume hybridization was performed at a temperature of 62 °C in multiplexes of 12 samples to increase complexity when quality control of the indexed libraries was < 100 ng total per sample. Quality controls and WES data analysis pipeline is described in Supplemental Methods.

### RRBS sequencing: data analysis

Reduced representation bisulphite sequencing (RRBS) raw reads were trimmed for adaptor sequences using trim galore (v. 0.6.5) (http://www.bioinformatics.babraham.ac.uk/projects/trim_galore/) and filtered for low-quality sequences using fastQC (v.0.11.8) (https://www.bioinformatics.babraham.ac.uk/projects/fastqc/). High quality trimmed reads were mapped to the Human reference genome (UCSC genome assembly GRCh38/hg38) using Bismark (v.0.22.3) [21] with default parameters. Methylation data as β values for CpG sites, promoters, and genes were retrieved from Bismark coverage outputs using R package RnBeads 2.0 (v.2.6.0) with default parameters [22]. RRBS data analysis process is described in Supplemental Methods.

### RNA extraction from FFPE sections, quality controls, RNA sequencing and data analysis

Total RNA was isolated from two to four 12 μm FFPE sections by RecoverAll Total Nucleic Acid Isolation Kit (Invitrogen), according to the manufacturer’s protocol including ethanol precipitation. RNA quantity and purity were estimated by a Nanodrop 2000 spectrophotometer (Thermo Fisher Scientific). Further quality controls (QCs) were performed on the TapeStation 4200 system using the RNA High Sensitivity ScreenTape Assay (Agilent Technologies). All samples had a concentration range between 5–10 ng/µl and RNA integrity number (RIN) between 2–5. The RNA exome analysis was performed at Center for Omics Sciences, IRCCS San Raffaele Scientific Institute, Milan, Italy, using sequence-specific capture of the coding regions of the transcriptome by Illumina RNA Prep with Enrichment (Illumina). To provide high reproducible sample handling RNA libraries were generated with epMotion® 5075 Liquid Handler (Eppendorf, Hamburg, Germany). All samples were sequenced on an Illumina NovaSeq 6000 sequencer in paired-end mode, generating 100 nt length reads, to obtain an average of 60 million clusters for RNA. Demultiplexing was performed using Illumina bcl2fastq2. Fastq quality was assessed using fastQC (v. 0.11.8) (https://www.bioinformatics.babraham.ac.uk/projects/fastqc/) and low-quality reads were discarded. Sequence reads were aligned to Human reference genome (UCSC genome assembly GRCh38/hg38) using STAR (v. 2.7.0b) [23], and the expression was quantified at gene level using featureCounts (v. 1.6.3), a count-based estimation algorithm [24]. Downstream analysis was performed in the R statistical environment as described in Supplemental Methods.

### Immunohistochemistry

Immunohistochemistry on formalin-fixed, paraffin-embedded (FFPE) tissues from human melanoma lesions was performed as described previously [25]. Briefly, 3 μm thick sections were stained with either Leica Bond RX stainer (Leica Biosystems, Buffalo, IL, USA) or Ventana BenchMark ULTRA immunostainer (Roche Diagnostics, Rotkreuz, Switzerland). The following antibodies were used: CD3 (Roche Diagnostics, clone 2GV6), CD4 (Roche Diagnostics, clone SP35). CD8 (Roche Diagnostics, clone SP57), CD68 (clone KP1, Dako Agilent, CA, USA), CD163 (Roche Diagnostics, clone MRQ-26), Fox-P3 (clone 236A/E7, Abcam, UK), PD-L1 (Dako Agilent, clone 22C3), PD-1 (clone NAT105, Biocare Medical, CA, USA), and HLA-Class I (Abcam, clone EMR8-5). EMR8-5 recognizes HLA Class I A,B,C and E antigens [26]. IHC data analysis is described in Supplemental Methods.

### Multiplex immunofluorescence (mIF) analysis of tumor microenvironment

Staining of 4–5 μm thick sections from FFPE sections was performed using the Leica Bond RX stainer (Leica Biosystems). The Bond RX staining protocol was implemented as described by Parra et al. [27]. Briefly, after baking and dewaxing with Bond Dewax solution (Leica Biosystems), slides were subjected to heating at 95 °C for 20 min in the presence of Bond Antigen Retrieval Tris–EDTA buffer or citrate buffer, depending on the markers. Slides were then incubated with the primary antibodies for 30 min. The following antibodies were used: CD8 (Leica Biosystems, clone 4B11), TCF-1/TCF7 (clone C63D9, Cell Signaling, MA, USA), TIM-3 (Cell Signaling, clone D5D5R) as well as PD-1 (clone EPR4877(2)) and S100/SOX10 (Clone EP268 1/4C4.9) ready-to-use antibodies all included in the Opal 6-Plex detection kit (Akoya Biosciences, Marlborough, MA, USA). DAPI was included for identification of nuclei. After wash and incubation with HRP following anti-mouse or anti-rabbit secondary antibodies slides were incubated with fluorophore tyramides of the Opal 6-Plex Detection Kit (Akoya Biosciences) containing the fluorophores Opal 480, Opal 520, Opal 620, Opal 690, Opal 780. After subsequent washes with Bond wash solution slides were counterstained with DAPI, to visualize nuclei. Slides were finally mounted with ProLong Diamond Antifade Mountain (Invitrogen). mIF data analysis is described in Supplemental Methods.

### Quantitative PCR analysis of melanoma cell lines

Melanoma cell lines (*n* = 46) were established, maintained and routinely tested for the absence of mycoplasma contamination by PCR, as previously described [28]. The tissue of origin of the cell lines and cell line authentication by STR profiling (Gene-Print10 kit, Promega) are described in Supplemental Table S5. Total RNA (0.5μg) was reverse transcribed with oligo d(t) using Superscript IV Reverse Transcriptase II (Invitrogen). Real-time PCR, in duplexing method, was carried out with 20ng input cDNA, 1X TaqMan Gene Expression Assays (Thermo Fisher Scientific) and Taqman Fast Advanced Master Mix (Thermo Fisher Scientific) on a Quant Studio 1 Real-Time PCR System (Applied Biosystems, Waltham, MA). The data were analysed by automated baseline and threshold determination. Relative expression was determined on triplicate reactions using the formula 2^−ΔCt^, reflecting target gene expression normalized to endogenous control level (GAPDH and GUSB). The following TaqMan assays were used:

- GAPDH_fam: GGGCGCCTGGTCACCAGGGCTGCTT (Hs99999905_m1);
- GUSB_vic: TGAACAGTCACCGACGAGAGTGCTG (Hs99999908_m1);
- MITF_fam: TCACAGAGTCTGAAGCAAGAGCACT (Hs01117294_m1);
- PMEL_vic: CATCTCTGATATATAGGCGCAGACT (Hs00173854_m1).

### Treatment with DNMTi of melanoma cell lines

Melanoma cell lines were seeded at 1.25 × 10^4^/mL in T75 flasks (Greiner Bio-One, Italy) with RPMI-1640 medium (Life Technologies/Thermo Fisher Scientific) supplemented with 4% FCS (Biological Industries/Sartorius AG, Gottingen, Germany) without antibiotics, and were treated with the DNMTi guadecitabine (MedChemExpress, D.B.A., Italy) according to a previously published protocol [29]. Briefly, cells were seeded at day 1, Guadecitabine was added at 1000 nM at days 2, 4, 8, 11, 15 and 18. Cells were harvested for gene expression and methylation profiling at day 7, or at day 14, or at day 21.

### Methylation analysis by infinium MethylationEPIC

Analyses were performed on melanoma cell lines treated or not with DNMTi, by Genomix4life S.R.L. (Baronissi, Salerno, Italy). DNA concentration in each sample was assayed with a fluorimeter Qubit Fluorometer 4.0 (Invitrogen), Nanodrop One (Thermo Fisher Scientific, Waltham, MA) and its quality assessed with the TapeStation 4200 (Agilent Technologies). Bisulfite converted DNA (250 ng) was used for analysis of whole-genome methylation using the Infinium MethylationEPIC v2.0 kit (Illumina), which contains ~  930 K unique methylation sites in the most biologically significant regions of the human methylome. In brief, bisulfite converted DNA was whole-genome amplified for 20 h followed by end-point fragmentation. Fragmented DNA was precipitated, denatured and hybridised to the BeadChips for 20 h at 48 °C. The BeadChips were washed and the hybridised primers were extended and labelled before scanning the BeadChips using the Illumina iScan system. GenomeStudio software (version 2011.1; Illumina) was used for the extraction of DNA methylation signals from scanned arrays. The methylation level for each cytosine was expressed as a beta value calculated as the fluorescence intensity ratio of the methylated to unmethylated versions of the probes: beta values ranged between 0 (unmethylated) and 1 (methylated).

### Statistical analysis

Integrative analysis of RNA-seq and RRBS data is described in Supplemental methods. Correlations between methylation subtypes and pathological variables were analysed using Pearson's Chi-squared Test. The Chi squared test was also used to compare the proportion of cases in each methylation class classified by the level of T cell infiltrate, stage progression, expression above or below the median z score value of gene signatures of interest, expression of immune markers and HLA class I antigens on tumor cells. The Kruskal Wallis test followed by Dunn’s multiple comparison test was used to compare methylation classes for expression of median z score values of gene signatures of interest. The Student T test was used to compare the expression of MITF, HMB45 and MART-1 proteins in cell lines treated or not with guadecitabine. Two-way ANOVA and Tukey’s multiple comparison test was used to compare the expression of melanoma differentiation signatures and the effects of guadecitabine treatment in melanoma clones. Survival curves were estimated using the survival R package (v. 3.2–10) and plotted using the Kaplan–Meier method, implemented in the survminer (v. 0.4.9) R package. Log-rank tests were used to compare survival curves between groups. Results of the multivariable Cox proportional hazards model on the TCGA skin cutaneous melanoma dataset were obtained through the outcome module of the TIMER2.0 web server (available at http://timer.comp-genomics.org). Differentially expressed genes for UR analysis among methylation-defined subsets were identified by BRB array tools with the following criteria: gene-level p value: 0.001; FDR < 0.01; permutation p value (based on 10,000 permutations): < 0.01. Lists of differentially expressed genes were used for UR analysis by Ingenuity Pathway Analysis, as described in Supplemental Methods.

## Results

### Clinical significance of DNA methylation classes in metastatic melanoma

**Fig. 1:**
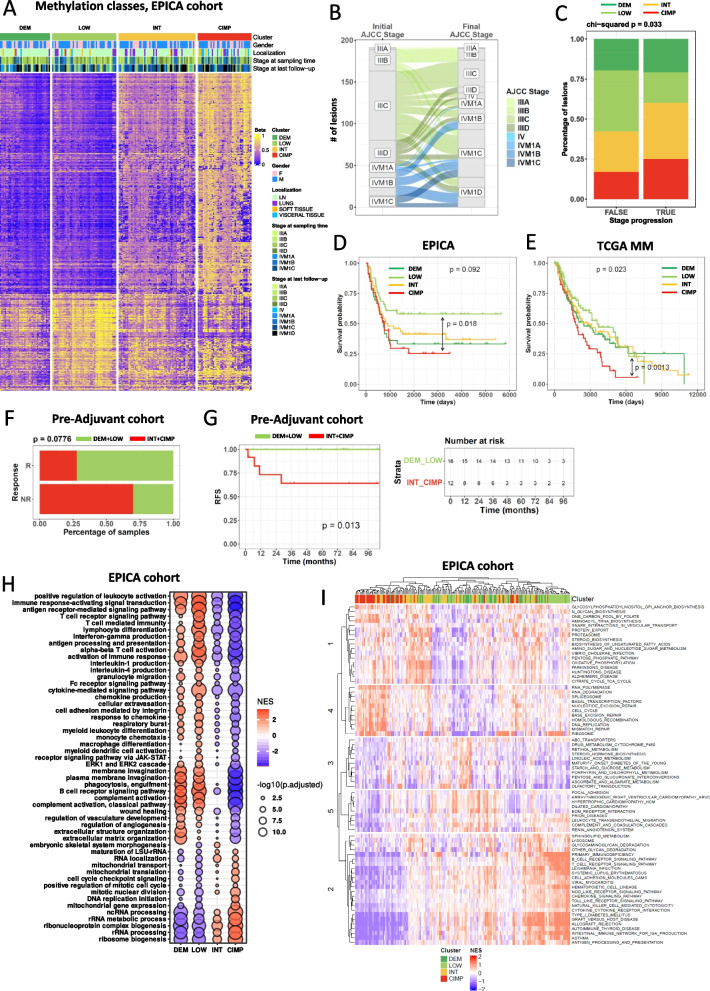
DNA Methylation profiling of MM EPICA cohort identifies four classes: clinical relevance and transcriptional programs. **A** Consensus clustering of *n* = 191 lesions from *n* = 165 AJCC stage III/IV MM patients (EPICA cohort) based on 4,064 most variable CpG sites. **B** Alluvial plot showing the initial AJCC stage of *n* = 165 patients in the EPICA cohort and the final AJCC stage at the end of follow-up. **C** Stacked bar plot showing the percentage of samples in EPICA cohort according to stage progression occurring (TRUE) or not (FALSE) after the surgical resection of the initial lesion used for methylation profiling. **D**, **E** Kaplan–Meier survival curves as a function of methylation cluster in EPICA cohort (**D**, *n* = 165) and in TCGA MM cohort (**E**, *n* = 368). Statistical analysis by Chi-square (**C**) and log rank test (**D**,** E**). **F** Bar plot for patients in an adjuvant ICB therapy cohort showing the percentage of pre-adjuvant samples in DEM/LOW vs INT/CIMP methylation groups, stratified by clinical outcome. Statistical significance by the chi-square test. **G** Kaplan–Meier relapse-free survival curves for patients in the pre-adjuvant cohort (*n* = 28), stratified by methylation class and grouped into DEM/LOW and INT/CIMP categories. The corresponding risk table is shown on the right. **H** Dot plot of the normalized enrichment score (NES) of significant Biological Processes (BP) obtained from GSEA (adjusted *p*-value < 0.05) in DEM, LOW, INT and CIMP methylation-defined classes of *n* = 187 lesions from *n* = 165 patients of the EPICA cohort. Dot color represent NES and the size represent *p*-value resulting from each comparison (one group vs all the others). GO terms selected are the top and bottom 25 significant for each comparison. **I** Unsupervised clustering of 191 samples of the EPICA cohort based on 72 KEGG pathways significantly enriched in at least the 80% of samples (absolute logit NES > 0.58 and *p*-value < 0.05)

We used reduced representation bisulfite sequencing (RRBS), RNA sequencing (RNA-seq) and whole exome sequencing (WES) on a fully annotated cohort of *n* = 191 melanoma lesions from *n* = 165 AJCC 8th edition stage III or IV patients (thereafter “EPICA cohort”) who underwent surgery for the resection of metastatic lesions between 09/2002 and 12/2017 (Supplemental Methods). Median follow-up was 28.4 months (range: 1.9 to 194.3 months from surgery for removal of the investigated lesions to death or to last follow-up) and 98.2% of the patients had not received any previous medical/systemic treatment (Supplemental Table S1A, B for all clinical and pathological data). By unbiased clustering of the top 1% most variable CpG sites (*n* = 4,064, Supplemental Table S3) we identified four classes with progressive increase in global methylation levels: demethylated (DEM), LOW, intermediate (INT) and hypermethylated (CIMP) (Fig. 1A). In the whole EPICA cohort the mean tumor purity, assessed by digital pathology on H&E-stained sections, was 83.1% (Supplemental Table S1B) and the CIMP class showed slightly higher tumor purity values compared to the DEM and LOW subsets (Supplemental Fig. S1A). We identified four methylation classes even in the TCGA Firehose Legacy melanoma cohort (www.cbioportal.org) of 368 MM lesions (Supplemental Fig. S1B) and in the TCGA cohort of 104 primary melanoma lesions (Supplemental Fig. S1C). In the EPICA cohort 103 out of 165 patients (62.4%) underwent further clinical stage progression during follow-up (Fig. 1B and Supplemental Table S1A). However, among patients who did not progress to any subsequent AJCC stage, 38.03% had LOW lesions, compared to 19.7%, 25.4% and 16.9% of non-progressors among patients with DEM, INT or CIMP lesions, respectively (Fig. 1C). The proportion of patients progressing to CNS metastases (AJCC Stage IVM1D) in the DEM, INT and CIMP clusters was respectively 25.0%, 17.3% and 26.5%, compared to 9.3% in the LOW cluster (Supplemental Table S1B). In both the EPICA and the TCGA MM cohorts, patients in the LOW and CIMP subsets showed the longest and the shortest survival, respectively (Fig. 1D, E). To test the potential relevance of DNA methylation classes in a therapeutic setting we used RRBS in an independent cohort of *n* = 28 pre-therapy lesions from Stage III-IV melanoma patients treated with adjuvant a-PD-1 and/or a-CTLA-4 ICB (Supplemental Table S2). The EPICA cohort samples were used as training set to classify the pre-adjuvant lesions into the DEM, LOW, INT and CIMP classes (Supplemental Table S2). We performed the subsequent analyses by merging the CIMP and INT, and the LOW and DEM subtypes. Based on the clinical outcome to adjuvant therapy, the CIMP + INT category was enriched among patients experiencing disease relapse (NR in Fig. 1F), although the trend did not reach statistical significance. However, crucially, patients belonging to the DEM + LOW category showed a strong and significant advantage in relapse free-survival compared to those in the INT + CIMP category (Fig. 1G).

Exome sequencing profiling of the EPICA cohort lesions revealed that mutations in the most frequently altered genes, such as BRAF and NRAS, were not associated with the methylation subsets (Supplemental Fig. S2). Only 6 genes (*RYR3, MGAM, SAMD9L, PAPPA2, TENM4, SNF208*) showed significant associations with the methylation classes. Moreover, the four methylation classes did not differ significantly in terms of the tumor mutational burden (TMB) value (Supplemental Fig. S2; *p* = 0.3 by Anova).

### Transcriptional landscape of DNA methylation subsets in metastatic melanoma

By integrating DNA methylation results with RNA-seq data in the EPICA cohort, we tested the relationship linking promoter methylation to gene expression in the four methylation clusters. By over-representation analysis we looked at distinct collections of gene sets (GO, HP, KEGG and REACT). In the LOW lesions, genes belonging to a large set of immune-related pathways were up-regulated and hypomethylated (Supplemental Fig. S3A), while genes belonging to developmental pathways were down-regulated and hypermethylated (Supplemental Fig. S3B). In contrast, genes belonging to cell–cell communication and developmental processes were up-regulated and hypomethylated in the DEM lesions (Supplemental Fig. S3A). In the CIMP lesions we found enrichment for down-regulated and hypermethylated gene sets belonging to immune-related pathways (Supplemental Fig. S3B). Therefore, immune-related pathways showed opposite methylation/expression relationships in the LOW vs CIMP classes. In agreement with these findings, by GSEA we found that the DEM and the LOW tumors were enriched for immune-related biological processes, while the CIMP and INT tumors were characterized by mitochondrial metabolism and cell cycle/proliferation pathways (Fig. 1H). An unsupervised pathway-based deconvolution algorithm [30] applied to 186 KEGG pathways, 72 of which were significantly enriched, segregated CIMP samples from LOW samples, identifying two extreme functional states confirming the association of cell cycle/proliferation pathways with CIMP and INT tumors and of immune-related pathways with LOW and DEM tumors (Fig. 1I).

### Immune-related master molecules with differential activation in DNA methylation classes control gene signatures with prognostic relevance

**Fig. 2:**
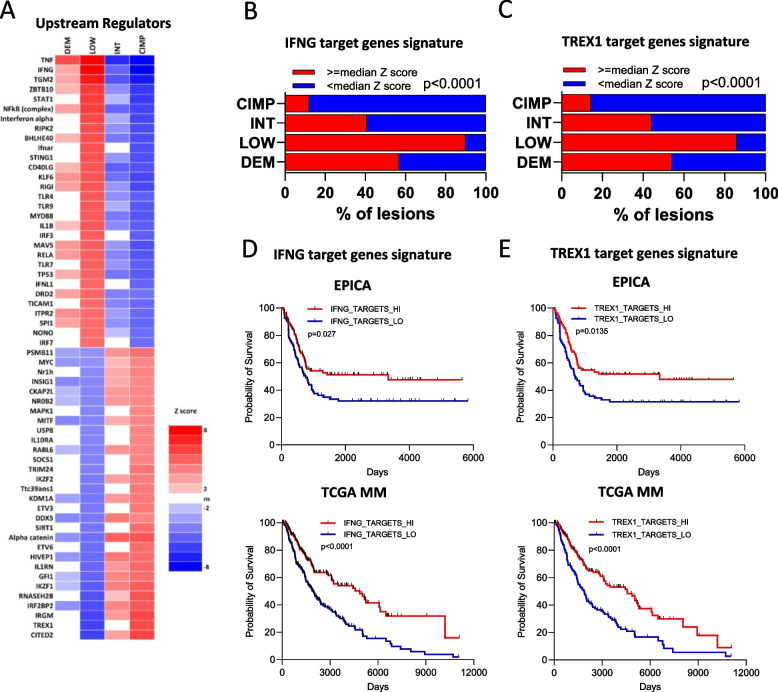
Top master molecules (Upstream Regulators) activated in LOW and CIMP MM classes regulate target gene signatures with prognostic significance. **A** Heatmap of IPA computed z scores for top UR predicted to be activated (red) or inhibited (blue) in each of the four methylation-defined clusters. **B**, **C** Stacked bar plots showing for each methylation cluster in the EPICA cohort the percentage of samples with expression of the IFNG (**B**) or TREX1 (**C**) target genes above or below the median z score value of each signature. **D**,** E** Kaplan–Meier survival curves of patients in the EPICA cohort (top plot) or TCGA MM cohort (bottom plot) according to median z score expression (from RNA-seq profiling) of IFNG (**D**) or TREX1 (**E**) target gene signatures. In **D** and **E**, patients in both cohorts were grouped according to median UR target gene expression above (“HI”) or below (”LO”) the median z score value of the signature. Statistical analysis **B**, **C** by Chi-square; in **D**, **E** by log rank test

We used Ingenuity Pathway Analysis (IPA) as described [29], on differentially expressed genes in the four methylation classes of EPICA (Supplemental Table S4). We found that the DEM and LOW subsets were characterized by activation of master molecules controlling innate and adaptive immunity (such as IFNG, TNF, IL1B), while the INT and CIMP subsets were characterized by inhibition of these regulators (Supplemental Fig. S4). By the IPA “Upstream Regulator” (UR) computational tool [29] we identified master molecules explaining the differences in gene expression among methylation subsets. LOW and CIMP lesions showed an opposite pattern of UR activation vs UR inhibition (Fig. 2A). In the LOW class top activated UR (e.g., IFNG, TNF, STAT1, NFkB, IFNA, STING1) were positive regulators of innate and adaptive immunity. In contrast, several top activated UR in CIMP subset (e.g., TREX1, IRGM, IL1RN, RNASEH2B, HIVEP1, SIRT1), were negative regulators of innate immunity pathways [31–35]. Activation of top UR as IFNG in LOW lesions reflected increased IFNG target gene expression in LOW compared to CIMP lesions (Supplemental Fig. S5). Conversely, due to their negative immune function, activation of top UR as TREX1, IRGM and IL1RN in CIMP lesions reflected reduced target gene expression in CIMP compared to LOW lesions (Supplemental Fig. S5). In agreement, the target gene signatures of these four UR were expressed above median z score value of each signature in the majority of LOW lesions, but below the median z score value in the majority of CIMP lesions (Fig. 2B, C and Supplemental Fig. S6A, C).

By using the “clinical outcome” module of the TIMER 2.0 web application, we found that the majority of the target genes of these four UR had prognostic significance in the TCGA MM dataset and conferred reduced risk (Supplemental Fig. S5). In agreement, we found that the target gene signatures of these four UR had prognostic significance in both the EPICA and TCGA MM cohorts (Fig. 2D, E and Supplemental Fig. S6B, D). We then extended this approach to test the prognostic significance of the target gene signatures of five additional top UR activated in CIMP (CITED2, HIVEP1, IRF2BP2, RNASEH2B and SIRT1) or activated in LOW lesions (TNF, TGM2, STAT1, NFkB and STING1). The target gene signatures of three of these UR (RNASEH2B, TNF, STING1) had prognostic significance in both the EPICA and TCGA MM cohorts (data not shown). Taken together, these findings suggest that distinct DNA methylation profiles in MM are associated with different transcriptional landscapes, which in turn influence clinical behavior.

### Association of MM methylation subsets with ICB response vs resistance gene signatures

**Fig. 3:**
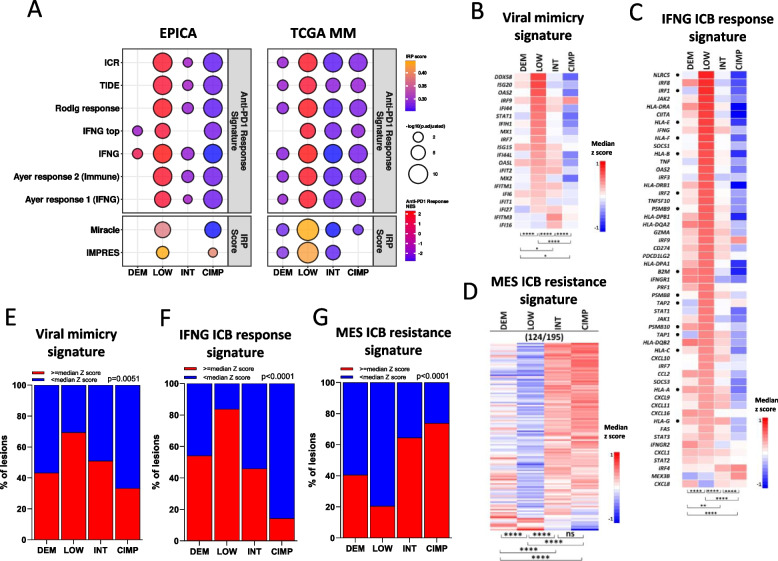
Expression of ICB predictive gene signatures in methylation-defined classes of the EPICA cohort. **A** top panels**:** dot plot of normalized enrichment scores (NES) from GSEA of seven anti-PD1 response signatures in the four methylation subsets of the EPICA and TCGA metastatic melanoma cohorts. Dot size represents the adjusted *p*-values and scale colors represent the NES (top). Bottom panels: dot plot of immune-response predictor scores (IRP) MIRACLE and IMPRES. Dot colors represent the mean score in each group and size the adjusted *p*-values (bottom). **B-D** Heatmaps of median z score expression of genes in the viral mimicry (**B**), IFN ICB response (**C**) and MES resistance (**D**) signatures in the four methylation subsets. Genes identified by dots in panel **C** represent a core HLA Class I APM gene set. **E**–**G** Stacked bar plots showing for each methylation cluster in the EPICA cohort the percentage of samples with expression of the viral mimicry (**E**), IFNG ICB response (**F**) and MES (**G**) signatures above or below the median z score value of each signature. Statistical analysis in **B-D** by Kruskal Wallis test followed by Dunn’s multiple comparison test; in **E**–**G** by chi square.*: *p* < 0.05; **: *p* < 0.01, ***: *p* < 0.001; ****: *p* < 0.0001

We asked whether the melanoma methylation subsets could be characterized by differential expression of predictive signatures of immunotherapy response. Indeed, two predictive scores, Miracle [36] and IMPRES [37] resulted in significantly different values and seven transcriptional signatures [38–42] were differentially expressed in the four methylation classes of both EPICA and TCGA MM cohorts (Fig. 3A). In the EPICA cohort the viral mimicry signature [43] and the IFNG ICB response signature [39] were enriched in the LOW compared to the DEM, INT and CIMP subsets (Fig. 3B-C, E–F). Conversely, the mesenchymal-like (MES) ICB resistance signature [44] was more expressed in the CIMP and INT subsets compared to the LOW and DEM classes (Fig. 3D, G). The results concerning the latter three signatures were validated in the methylation-defined classes of the TCGA MM cohort (Supplemental Fig. S7A-F).

### An inflamed, T cell rich tumor microenvironment with high frequency of intra-tumor CD8+TCF-1+ PD-1+ TIM-3− pre-exhausted T cells characterizes LOW lesions compared to CIMP tumors

We used deconvolution algorithms, immunohistochemistry (IHC) and multiplex immunofluorescence to investigate in detail the tumor immune microenvironment of the methylation subsets in the EPICA cohort. Melanoma microenvironment-specific signatures [45, 46] and Xcell algorithm [47] indicated that the LOW and the CIMP subsets were, respectively, the most and the least enriched for B cells and T cells at distinct stages of functional differentiation, as well as for myeloid or plasmacytoid dendritic cells (Supplemental Fig. S8A, B). Compared to DEM, INT and CIMP subsets, the LOW lesions were enriched for expression of gene signatures defining pro-tumoral macrophages [48], tertiary lymphoid structures [49], pre-exhausted T cells (T_PEX_) [50–52] and exhausted T cells (T_EX_) [52, 53] (Supplemental Fig. S8C-H). These findings were validated in the four methylation classes of the TCGA MM dataset (Supplemental Fig. S8A and data not shown).

**Fig. 4:**
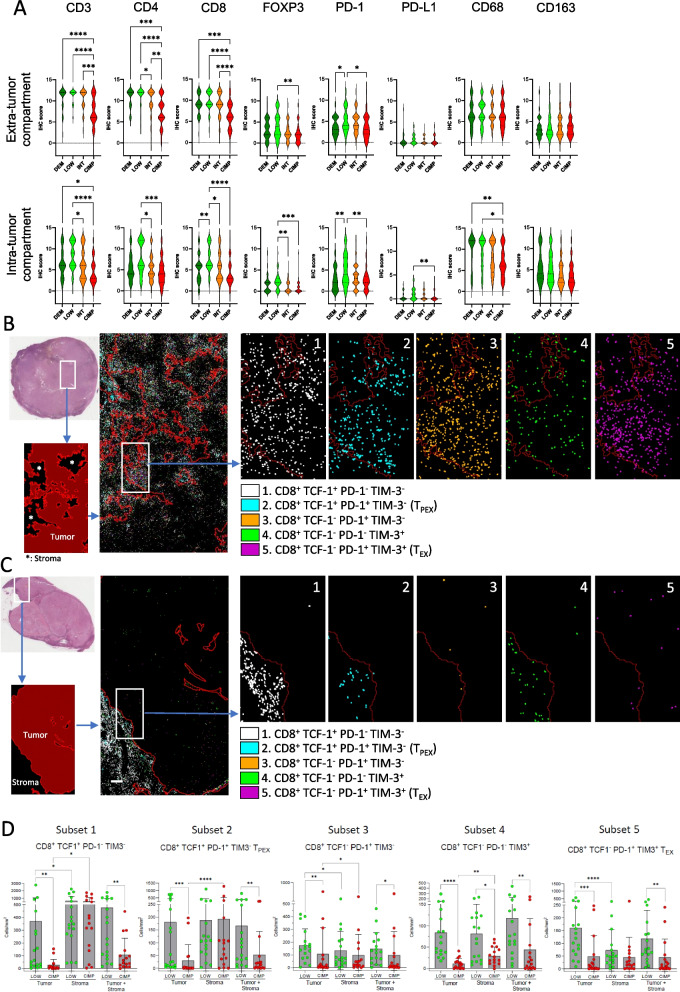
Infiltrating T cells, T_PEX_ and T_EX_ are enriched in LOW melanomas compared to CIMP melanomas. **A** Violin plots showing expression, by semi-quantitative IHC, of CD3, CD4, CD8, PD-1, PD-L1, CD68 and CD163 in extra-tumor or intra-tumor compartments of *n* = 191 EPICA lesions classified according to DEM (*n* = 39), LOW (*n* = 50), INT (*n* = 60), CIMP (*n* = 42) methylation classes. Data expressed as IHC score (see Supplemental Methods). **B**,**C** Multiplex immunofluorescence analysis of a representative LOW (**B**) and CIMP (**C**) lesions. In **B** and **C**, the H&E image (top) shows the area used for tissue segmentation (bottom) with tumor and stroma identified in dark red and black, respectively. A higher magnification field of the same area shows the density and position of 5 main CD8^+^ T cell phenotypes identified based on differential expression of TCF-1, PD-1 and TIM-3 and color-coded as indicated. Visualization of tumor cells was omitted in **B**,**C**. **D** Density (cells/mm^2^) in tumor, stroma and whole tissue (tumor + stroma) of the 5 CD8^+^ subsets defined by differential expression of TCF1, PD-1 and TIM-3 in LOW (*n* = 17) and CIMP (*n* = 16) lesions. Statistical analysis: in **A** by Kruskal Wallis test followed by Dunn’s multiple comparison test; in **D**, by Mann Whitney test for LOW vs CIMP comparisons in each microenvironment compartment and by Friedman multiple comparison test for tumor vs stroma vs tumor + stroma comparisons within each methylation subset. *: *p* < 0.05; **: *p* < 0.01; ***: *p* < 0.001; ****: *p* < 0.0001

We then used IHC to stain EPICA lesions (*n* = 191) for CD3, CD4, CD8, PD-1, PD-L1, FOXP3, CD68 and CD163. The LOW group, when compared to the CIMP subset, showed the highest scores for CD3, CD4, CD8, FOXP3 and PD-1 in intra-tumor and extra-tumor compartments (Fig. 4A). PD-L1 and CD68 had higher IHC scores in LOW vs CIMP lesions in the intra-tumor compartment, (Fig. 4A). The LOW subset was enriched for tumors with a strong intra-tumor infiltrate of CD3^+^ T cells while the CIMP subsets was enriched for lesions with an excluded CD3^+^ and CD8^+^ infiltrate (Supplemental Fig. S9A, B). Compared to patients with CIMP tumors, a larger fraction of patients with LOW tumors and with higher-than-median CD3, CD4 and CD8 IHC scores did not undergo further clinical stage progression during follow-up (Supplemental Fig. S10, dark blue bars).

We then asked whether LOW vs CIMP tumors could show significant differences for presence of CD8^+^ T cell subsets at different stages of functional differentiation, including pre-exhausted T cells (T_PEX_)_,_ a subset associated with responsiveness to ICB [54]. To this end we used multiplex immunofluorescence analysis (Supplemental Fig. S11A-D for analysis strategy) on sections stained for CD8, TCF1, PD-1 and TIM-3. This approach allowed to identify 5 CD8^+^ stages, from the less differentiated CD8^+^ TCF1^+^ cells, including the TCF1^+^ PD-1^+^, TIM-3^−^, a profile consistent with T_PEX_ [55], to the more differentiated TCF1^−^, PD-1^+^, TIM-3^+^ phenotype, consistent with T_EX_ [56]. All five CD8^+^ subsets including T_PEX_ and T_EX_ (subsets 2 and 5, in Fig. 4B-D) showed significantly higher cell densities in tumor areas of the LOW lesions compared to CIMP lesions (Fig. 4B-D). Taken together, these results indicate that LOW and CIMP lesions have divergent immune contextures, with the LOW subset enriched for T cell inflamed lesions and for presence of CD8^+^ subsets at different stages of functional differentiation.

### A main mechanism of immune escape is associated with the MM methylation classes

Fifteen genes (black dots close to the gene symbols in Fig. 3C) in the IFNG ICB response signature, represent the core of the HLA class I antigen processing and presentation pathway. A low expression of this gene signature in a tumor lesion may potentially reveal a mechanism of immune escape, i.e. downmodulation/loss of HLA class I molecules on tumor cells. To test this hypothesis, we first set out to identify the most informative genes in the HLA class I pathway. To this end we combined the 15 genes of the IFNG ICB signature with 25 additional genes, selected through literature search, and involved in the HLA class I pathway and in its positive and negative regulation. By spearman correlation analysis of the expression levels we found that 19 out of 40 genes (gene set 2 in Supplemental Fig. S12A, thereafter “HLA-I APM signature”) were directly and significantly correlated in both EPICA and TCGA MM cohorts (Supplemental Fig. S12A). The HLA Class-I APM signature was expressed above median z score values in a higher fraction of LOW compared to CIMP tumors in the EPICA cohort (Supplemental Fig. S12B) and showed prognostic significance in the EPICA and TCGA MM cohorts (Supplemental Fig. S12C).

**Fig. 5:**
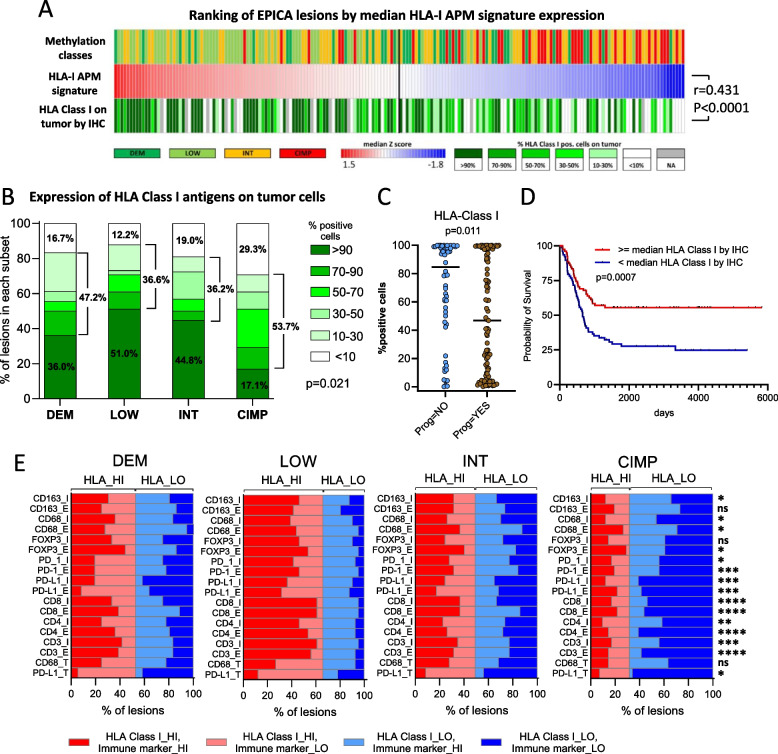
Downmodulation/loss of expression of HLA class I molecules on tumor cells is more frequent in CIMP compared to LOW lesions. **A** Ranking of EPICA lesions (*n* = 175), colored by methylation subset, according to median expression z score (from RNA-seq profiling) of the HLA Class I APM signature and according to expression of HLA Class I molecules on tumor cells by IHC. **B** Stacked bar plots showing for each methylation cluster in the EPICA cohort the percentage of samples in each of six classes of HLA Class I antigen expression on tumor cells (by IHC and quantitative digital pathology analysis). **C** Association of HLA class I antigen expression on tumor cells in the EPICA cohort with progression to any subsequent AJCC stages. **D** Kaplan–Meier survival analysis of patients in the EPICA cohort according to expression of HLA Class I antigens on tumor cells. **E** Association of methylation classes with immune contexture and with expression of HLA Class I molecules on tumor cells. Expression of HLA Class I and of immune markers, by IHC, was dichotomized into “HI” and “LO” groups corresponding to expression above or below the median value of each parameter. Statistical analysis in **A** by Spearman correlation; in **B**,**E** by Chi-square; in **C** by Mann–Whitney test, in **D** by log rank test. *: *p* < 0.05; **: *p* < 0.01; ***: *p* < 0.001; ****: *p* < 0.0001

We then used IHC, coupled to quantitative digital pathology analysis in the EPICA cohort (*n* = 175 lesions) to directly assess expression of HLA Class I molecules on tumor cells. Expression of HLA Class I antigens on tumor cells by IHC and of the HLA Class-I APM gene signature were positively correlated in the whole EPICA cohort (*r* = 0.431, *p* < 0.0001, Fig. 5A). Lesions retaining high expression of HLA Class I or showing variable levels of HLA Class I downmodulation were found in all four methylation subsets (Fig. 5A and Supplemental Fig. S13 for representative stainings). However, lesions expressing HLA class I molecules on > 90% of the tumor cells were 51% in the LOW subset, compared to 17.1% in the CIMP class (Fig. 5B). Conversely, lesions with almost complete HLA Class I downmodulation (< 10% of positive tumor cells) were 29.3% in the CIMP subset compared to 12.2% of the LOW subset (Fig. 5B). In the EPICA cohort, high expression of HLA class I molecules on tumor cells was associated with lack of further AJCC stage progression (Fig. 5C) and with improved survival (Fig. 5D). Moreover, the LOW subset contained the highest proportion of “HLA Class I^HI^/immune infiltrate^HI^” lesions compared to the CIMP subset (Fig. 5E). Conversely, CIMP subset contained the highest proportion of “HLA Class I^LO^/immune infiltrate^LO^” lesions compared to the LOW ones (Fig. 5E). Collectively, these data indicate preferential downmodulation/loss of expression of HLA Class I molecules on tumor cells in CIMP compared to LOW lesions and highlight the clinical relevance of HLA Class I downmodulation in MM.

### CIMP lesions are enriched for proliferative, differentiated melanomas, while LOW lesions are enriched for de-differentiated melanomas

To test the association of methylation subsets with melanoma biological features and differentiation programs we compared expression of four distinct gene signatures in DEM, LOW, INT and CIMP subsets of EPICA cohort. The melanoma-specific cell cycle (MCC) signature [45] was enriched in the CIMP lesions compared to all other subsets (Supplemental Fig. S14A). The IFNG de-differentiation (IFNG-dediff) signature [57], unrelated to the IFNG ICB response signature, and the TEADS invasive signature [58], were strongly enriched in the LOW and DEM subsets compared to the INT and CIMP subsets in both the EPICA and TCGA MM cohorts (Supplemental Fig. S14B-E). We then tested expression in the EPICA methylation subsets of the 7 melanoma differentiation sub-signatures described by Tsoi et al. [59]. The “melanocytic” and “transitory-melanocytic” sub-signatures were more expressed in CIMP and INT tumors. The “neural crest-like”, “undifferentiated/neural crest-like” and “undifferentiated” sub-signatures were more expressed in the LOW compared to the CIMP lesions (Supplemental Fig. S14F). Taken together, these data indicate that the DNA methylation class is associated with the melanoma differentiation phenotype: CIMP tumors are enriched for differentiated, proliferative melanomas, while LOW lesions are enriched for de-differentiated melanomas.

### The differentiation and immune-related transcriptional profiles observed in CIMP vs LOW lesions are tumor-intrinsic programs retained in-vitro by melanoma cell lines

**Fig. 6:**
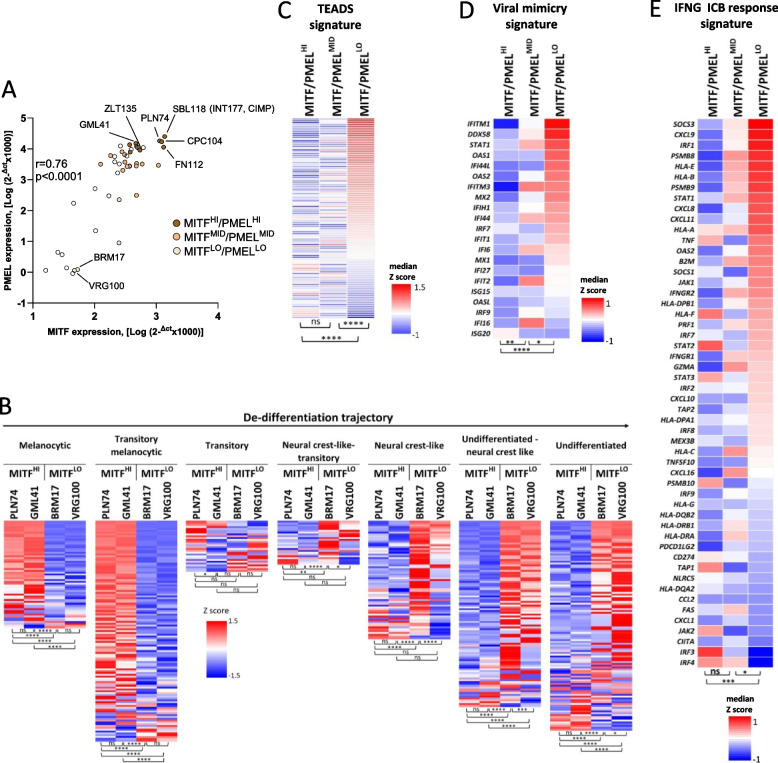
Differential expression of melanoma differentiation signatures and of immunotherapy response signatures in MITF/PMEL^HI^ vs MITF/PMEL^LO^ cell lines. **A** Expression of MITF and PMEL genes, by qPCR, in 46 melanoma cell lines. Subsets of cell lines with high (HI), intermediate (MID) and low (LO) MITF/PMEL expression are highlighted by the indicated color code. Cell line SBL118 was generated from the surgical specimen of the lesion corresponding to sample INT177 of the EPICA cohort. **B** Heatmaps showing expression of the 7 melanoma differentiation sub-signatures described by Tsoi et al. [59] in four melanoma cell lines. **C**,**D**,**E** Heatmaps showing expression of the TEADS invasive signature (**C**), of the viral mimicry (**D**) and of the IFNG-ICB response signatures (**E**) in three sets of melanoma cell lines (MITF/PMEL^HI^, MITF/PMEL^MID^, MITF/PMEL.^LO^). Statistical analysis in **A** by Spearman correlation; in **B** by two-way anova followed by Tukey’s multiple comparison test; in **C**, **D**, **E** by Kruskal Wallis test followed by Dunn’s multiple comparison test. *: *p* < 0.05; **: *p* < 0.01; ***: *p* < 0.001, ****: *p* < 0.0001

We used a panel of 46 melanoma cell lines (*n* = 45 from metastatic lesions, *n* = 1 from a primary tumor) derived from surgical specimens of patients (Supplemental Table S5 for cell line origin and STR profiling authentication). By qPCR for two markers of differentiated melanomas (MITF and PMEL) we identified MITF/PMEL^HI^, MITF/PMEL^INT^ and MITF/PMEL^LO^ cell lines (Fig. 6A). Transcriptomic analysis revealed higher expression of the “melanocytic” and “transitory-melanocytic” sub-signatures [59], in the MITF/PMEL^HI^ cell lines (PLN74 and GML41), and higher expression of the “neural crest-like”, “undifferentiated-neural crest-like” and “undifferentiated” sub-signatures in two MITF/PMEL^LO^ cell lines (BRM17 and VRG100, Fig. 6B). Consistently, expression of the TEADS invasive signature was highest in the MITF/PMEL^LO^ cell lines compared to MITF/PMEL^MID^ and MITF/PMEL^HI^ cell lines (Fig. 6C). Crucially, the viral mimicry and the IFNG-ICB response signatures were expressed at higher levels in the de-differentiated MITF/PMEL^LO^ cell lines compared to the differentiated MITF/PMEL^HI^ cell lines (Fig. 6D, E), mirroring the different profiles observed in LOW vs CIMP lesions, respectively.

### Treatment of differentiated melanoma cell lines with a DNMTi induces global DNA de-methylation, promotes de-differentiation and upregulates ICB predictive signatures

**Fig. 7:**
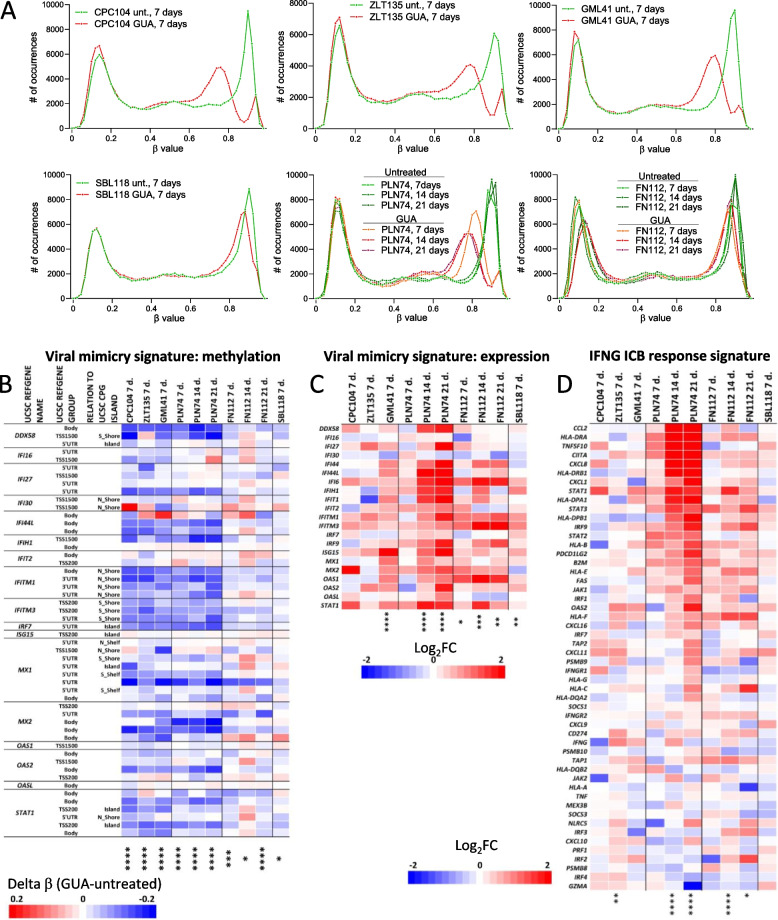
DNA de-methylation, promoted by a DNMTi, shifts the transcriptional profile of differentiated melanoma cell lines towards the “immune-high” phenotype found in LOW lesions. **A** Global DNA methylation profiles, from EPIC array data, of six differentiated melanoma cell lines untreated or treated with guadecitabine (GUA) for 7 to 21 days. **B** Changes in methylation level induced by guadecitabine treatment at methylation sites corresponding to genes in the viral mimicry signature in 6 differentiated melanoma cell lines cultured with guadecitabine for 7 to 21 days. **C**,**D** Treatment with guadecitabine for 7 to 21 days of six melanoma cell lines modulates genes in the viral mimicry signature (**C**) and in the IFNG ICB response signature (**D**). Statistical analysis in **C**-**E** by Mann–Whitney test. *: *p* < 0.05; **: *p* < 0.01; ***: *p* < 0.001; ****: *p* < 0.0001

Six MITF/PMEL^HI^ differentiated melanoma cell lines were treated with the DNMTi guadecitabine for 7 to 21 days, according to a previously published protocol [29]. Based on the extent of global DNA de-methylation, measured by EPIC arrays in guadecitabine-treated vs controls, four out of six cell lines (CPC104, ZLT135, GML41, PLN74) were highly responsive to this DNMTi, (Fig. 7A). In the responsive cell line PLN74, global DNA de-methylation promoted by guadecitabine increased at 14 and 21 days, compared to 7 days of treatment (Fig. 7A). Two cell lines (SBL118 and FN112) were weakly responsive to the DNMTi, as they showed only modest reduction in global DNA methylation levels at day 7 of treatment, with no further decrease at 14 and 21 days (cell line FN112, Fig. 7A). By analysis of Clariom S transcriptomic data in the highly responsive PLN74 cell line, we found that genes in the proliferative MCC signature [45] and in “melanocytic” and “transitory-melanocytic” sub-signatures [59] were downmodulated by guadecitabine treatment (Supplemental Fig. S15A). In contrast, genes in the TEADS [58] and IFNG-dediff signatures [57], as well as genes in the “neural crest-like”, “undifferentiated-neural crest-like” and “undifferentiated” sub-signatures [59] were progressively upregulated (Supplemental Fig. S15A). In the same PLN74 cell line, guadecitabine treatment strongly downmodulated expression of three differentiation proteins (MITF, GP100/PMEL, MART-1), as evaluated by quantitative digital pathology on melanoma cell cytospins (Supplemental Fig. S15B, D). In contrast, markedly reduced effects were observed on the expression of these three markers in the poorly responsive FN112 cell line (Supplemental Fig. S15C, E). We then tested guadecitabine effects on melanoma clones isolated from the same metastatic tumor 665/2 by cloning in soft agar and micromanipulation [60]. Based on the Tsoi et al. differentiation sub-signatures [59] clones 2_21, 2_33 and 2_59 exhibited a differentiated profile, while clones 2_4, 2_14 and 2_17 showed a predominant de-differentiated profile (Supplemental Fig. S16A). Upon treatment for 7 days with guadecitabine, the differentiated melanoma clone 2_59 underwent a process of de-differentiation (Supplemental Fig. S16B), as shown by downmodulation of “melanocytic” and “transitory-melanocytic” sub-signatures and by upregulation of “neural crest-like”, “undifferentiated-neural crest-like” and “undifferentiated” sub-signatures [59]. No consistent profile shift was observed in the de-differentiated clone 2_17.

We then evaluated the impact of guadecitabine treatment on the methylation and expression of genes belonging to immunotherapy response signatures. Strong de-methylation at sites corresponding to genes in the viral mimicry signature was observed (Fig. 7B) in four highly responsive cell lines (CPC104, ZLT135, GML41, PLN74). Upregulation of genes in this signature was also observed in four cell lines and most significant effects were induced in PLN74 cells at day 14 and day 21 of treatment (Fig. 7C). In 3/6 melanoma cell lines significant upregulation of the genes in the IFNG-ICB response signature was also observed upon guadecitabine treatment (Fig. 7D).

Finally, we selected the top UR found constitutively activated or inhibited in CIMP lesions (shown in Fig. 2A) and tested whether the functional status of these UR could be reversed in differentiated melanoma cell lines (PLN74, GML41, CPC104, FN112 and SBL118) upon treatment with guadecitabine. In all these cell lines we found that DNMTi treatment activated the UR found inhibited in CIMP tumors and inhibited the UR found activated in CIMP lesions (Supplemental Fig. S17). Taken together, these results indicate that global DNA de-methylation, induced by a DNMTi, profoundly reshapes the transcriptomic profile of differentiated melanoma cell lines towards a de-differentiated and “immune-high” phenotype mirroring that observed in LOW tumors.

## Discussion

In this study, by exploiting epigenetic variability at the methylome level, we identified four methylation subsets in the EPICA and TCGA MM cohorts. Extensive multi-omics characterization of the four subsets revealed that the epigenetic classification provides a framework for explaining several layers of MM complexity including: transcriptional programs of melanoma differentiation and immunity, prognostically relevant gene signatures controlled by master molecules, expression of ICB response and resistance signatures, specific immune microenvironment structure, a main immune escape mechanism, propensity for subsequent stage progression and clinical outcome. The LOW and the CIMP tumors exhibited the most divergent phenotypic profiles and appear to represent opposite ends of the spectrum of melanoma biological heterogeneity. The enrichment, in tumors of the LOW class, for prognostically relevant signatures and for lesions with a strong T cell infiltrate, suggests that this subset contains a high proportion of metastatic lesions with an active anti-tumor response. Such immune response may contribute to reduce the likelihood of further clinical stage progression and to improve overall survival, as we found in patients with LOW tumors compared to patients with CIMP lesions. The high expression of ICB predictive signatures and the enhanced density at tumor site of CD8^+^ T cell subsets known to be associated with response to immunotherapy (such as the T_PEX_), suggests that patients with LOW lesions might have increased responsiveness to ICB. In support of this hypothesis, in an independent adjuvant ICB cohort, we found that patients with DEM/LOW pre-therapy lesions had a significantly better relapse-free survival compared to patients with INT/CIMP lesions. These findings suggest that the DNA methylation profile of neoplastic lesions could be exploited to predict which MM patients may benefit or show resistance to ICB therapy. Indeed, at least two recent studies [61, 62] in the metastatic setting support this conclusion. In one of these studies [62], three methylation subsets were identified. Cluster 1, the most hypomethylated, contained only responders, cluster 2 contained 50% of responders while the hypermethylated cluster 3 contained 39 non-responders out of 45 patients in the cluster. In the adjuvant cohort we also observed that the CIMP + INT category was enriched among patients experiencing disease relapse, but the trend did not reach statistical significance. Although transcriptomic and immune contexture data were not available in such cohort, we cannot rule out the possibility that the INT + CIMP category of the adjuvant cohort may show defective response to adjuvant immunotherapy through some of the mechanisms that we found associated with the CIMP subset in the EPICA cohort. The metastatic lesions surgically removed before adjuvant treatment from the INT/CIMP category might in principle show an excluded or desert immune contexture, increased expression of immunotherapy resistance signatures and/or defective expression of HLA class I molecules on tumor cells.

In EPICA cohort, compared to LOW lesions, CIMP tumors showed a differentiated, proliferative profile, higher expression of an ICB resistance signature, higher frequency of lesions with HLA Class I downmodulation/loss on neoplastic cells, a frequently cold immune microenvironment and worse overall survival. In agreement with our findings, one of three methylation subtypes identified among 50 MM by Lauss et al. [15] showed elevated methylation at promoter islands and consisted of “proliferative” and “pigmentation type” tumors, thus resembling the differentiated CIMP subset defined in this study. The evidence for worse clinical outcome in the CIMP vs the LOW class, that we found in both the EPICA and TCGA MM cohorts, is to be considered in the light of similar evidence obtained by Conway et al. in primary melanomas [14]: collectively these findings suggest that acquisition of the hypermethylated profile “imprints” a poor clinical outcome across all stages of melanoma progression. In agreement, in the recent study [17] the authors found a worse outcome in Stage II and III melanoma patients whose primary tumors were classified in the CIMP methylation class.

The experiments with a panel of melanoma cell lines allowed us to test the cause-effect relationship linking the global DNA methylation profile of melanoma cells to their transcriptional programs. First, we found that differentiated and de-differentiated melanoma cell lines showed respectively lower vs higher expression of the viral mimicry and ICB response signatures. These findings suggested that level of constitutive expression of clinically relevant immune-related signatures is a cell autonomous feature of melanoma cells associated with their melanocyte differentiation programs. Second, promotion of global DNA de-methylation by the DNMTi guadecitabine shifted the phenotype of differentiated melanoma cells towards a de-differentiated and “immune-high” phenotype. Taken together these findings support the notion that global DNA methylation profile of MM cells contributes to shape the overall transcriptional phenotype of the tumor. Moreover, these findings provide a pre-clinical rationale supporting use of demethylating agents to promote *in-vivo* the rewiring of melanoma transcriptional programs towards an immunotherapy responsive profile. Results of the multi-omics analysis of pre- and post-therapy lesions from MM patients enrolled in our Phase 1b NIBIT-M4 trial with guadecitabine + ipilimumab support this notion [63]. Although that trial was not designed to compare the effect of a demethylating agent plus ICB vs ICB alone, post-therapy lesions from responding patients showed promotion of expression of ICB response signatures and a significant anti-correlation between methylation and expression of LINE, SINE and LTR elements, including ERVs [63]. These data suggest that a demethylating agent may, through global demethylation, reshape the transcriptional programs of melanoma cells in-vivo and activate transcription of ERV sequences, that in turn trigger the viral mimicry process crucial for ICB response [43].

The sensitizing effect of demethylating agents on immune checkpoint inhibitors is supported by additional evidence and further mechanisms. A guadecitabine-specific gene signature, that we recently identified in-vitro in melanoma cell lines treated with this DNMT inhibitor [29], was selectively activated in on-treatment tumor biopsies of responding patients compared to non-responding ones in the NIBIT-M4 trial [63]. This indicates that clinical response to ICB+DNMTi is promoted if tumor lesions upregulate the same set of immune-related genes modulated in vitro in neoplastic cells by the DNMTi. By transcriptomic analysis of NIBIT-M4 trial tumor lesions [63] we also found that several B cell-, TLS-, T_EX_- and IFNG-related gene signatures were selectively upregulated in on-treatment lesions from responding patients compared to non-responding ones. Interestingly, in this study we found that two of these signatures, predicting response to ICB and described by Chiappinelli et al. [43] and by Grasso et al. [39], were upregulated by guadecitabine in-vitro in melanoma cell lines and were constitutively upregulated in LOW lesions compared to CIMP tumors. Fittingly, genes belonging to ICB predictive signatures have been found upregulated in post-treatment tumor lesions from ovarian cancer patients treated with guadecitabine + a-PD-1 [64], as well as in T cell lymphoma lines [65] and in urothelial carcinoma cell lines [66] treated in-vitro with DNMTi. Finally, several studies with murine models [67–71] have shown that demethylating agents promote response to immune chekpoint inhibitors by at least two mechanisms: a) modulation of sets of immune-related genes crucial for the adaptive response to cancer cells; b) reshaping of the structure of tumor microenvironment in favor of an immune contexture enriched for functional T cells.

## Supplementary Information

Additional file 1. Supplemental Methods.docx

Additional file 2: Supplemental Figures_S1_to_S17.PDF. Fig. S1. Tumor purity in EPICA methylation subsets and validation of methylation classes in Firehose Legacy TCGA primary and metastatic melanoma cohorts. Fig. S2. Oncoplot displaying common somatic nonsynonymous alterations in the Stage III/IV EPICA MM cohort, divided by methylation groups. Fig. S3. Promoter methylation vs. gene expression analysis. Fig. S4. IPA core analysis for differentially expressed genes in the DEM, LOW, INT and CIMP EPICA classes. Fig. S5. Expression and prognostic significance of the IFNG, TREX1, IRGM, and IL1RN target genes in EPICA methylation classes. Fig. S6. Top master molecules activated in CIMP MM classes are negative regulators of target genes with prognostic significance. Fig. S7. Expression of ICB predictive signatures in methylation-defined classes of the TCGA MM cohort. Fig. S8. Assessment of immune-related gene signatures in the EPICA methylation-defined clusters. Fig. S9. LOW lesions are enriched for intra-tumor T cells compared to CIMP lesions. Fig. S10. Association of methylation classes and tumor immune contexture with subsequent stage progression in EPICA cohort. Fig. S11. Analysis strategy for identification of CD8^+^ subsets characterized by differential expression of TCF-1, PD-1 and TIM-3 in EPICA melanoma lesions by multiple immunofluorescence (mIF). Fig. S12. Correlation analysis in EPICA and TCGA MM cohorts of genes in the HLA Class I APM pathway and clinical significance of the HLA Class I APM signature. Fig. S13. Expression of HLA Class I antigens on tumor cells in representative lesions belonging to the DEM, LOW, INT and CIMP classes of the EPICA cohort. Fig. S14. Expression of melanoma differentiation signatures in EPICA methylation subsets. Fig. S15. Guadecitabine treatment promotes melanoma de-differentiation. Fig. S16. Guadecitabine promotes de-differentiation of the differentiated melanoma clone 2_59. Fig. S17. Guadecitabine treatment of differentiated melanoma lines reverses the activation state of UR identified in CIMP lesions.

Additional file3: Supplemental Tables_S1_to_S5.xslx. Table S1A. Summary of EPICA cohort clinical and pathological data. Table S1B. EPICA cohort, lesion centric data. Table S2. Adjuvant immunotherapy cohort, patient centric data. Table S3. Variable CpG sites in EPICA cohort. Table S4. Differentially expressed genes among methylation classes in EPICA cohort. Table S5. Origin and cell line authentication by STR profiling of melanoma cell lines

## Data Availability

The processed WES, RRBS, RNA-seq, Clariom S and microarray data generated in this study have been deposited on Zenodo repository under DOI 10.5281/zenodo.15584210.

## Abbreviations

AJCC: American Joint Committee on Cancer
CIMP: CpG island methylator phenotype
DEM: Demethylated
DIN: DNA integrity number
DNMTi: DNA methyl transferase inhibitor
FFPE: Formalin-fixed, paraffin-embedded
GSEA: Gene set enrichment analysis
ICB: Immune chekpoint bockade
IFNG: Interferon γ
IHC: Immunohistochemistry
INT: Intermediate
IPA: Ingenuity Pathway Analysis
MCC: Melanoma-specific cell cycle signature
MES: Mesenchymal-like
mIF: Multiplex immunofluorescence
MM: Metastatic melanoma
QPCR: Quantitative polymerase chain reaction
RIN: RNA integrity number
RNA-seq: RNA-sequencing
RRBS: Reduced representation bisulfite sequencing
TCGA: The Cancer Genome Atlas
T_EX_: Exhausted T cells
TMB: Tumor mutational burden
T_PEX_: Pre-exhausted T cells
UR: Upstream Regulator
WES: Whole exome sequencing
