# Supplementary material for: Integrated multi-omics profiling reveals the role of the DNA methylation landscape in shaping biological heterogeneity and clinical behaviour of metastatic melanoma
Source: J Exp Clin Cancer Res. 2025 Jul 18;44:212. doi: 10.1186/s13046-025-03474-9 (PMC12273276; doi:10.1186/s13046-025-03474-9)
Supplement: Supplementary file 2 — Additional file 2: Supplemental Figures_S1_to_S17.PDF. Fig. S1. Tumor purity in EPICA methylation subsets and validation of methylation classes in Firehose Legacy TCGA primary and metastatic melanoma cohorts. Fig. S2. Oncoplot displaying common somatic nonsynonymous alterations in the Stage III/IV EPICA MM cohort, divided by methylation groups. Fig. S3. Promoter methylation vs. gene expression analysis. Fig. S4. IPA core analysis for differentially expressed genes in the DEM, LOW, INT and CIMP EPICA classes. Fig. S5. Expression and prognostic significance of the IFNG, TREX1, IRGM, and IL1RN target genes in EPICA methylation classes. Fig. S6. Top master molecules activated in CIMP MM classes are negative regulators of target genes with prognostic significance. Fig. S7. Expression of ICB predictive signatures in methylation-defined classes of the TCGA MM cohort. Fig. S8. Assessment of immune-related gene signatures in the EPICA methylation-defined clusters. Fig. S9. LOW lesions are enriched for intra-tumor T cells compared to CIMP lesions. Fig. S10. Association of methylation classes and tumor immune contexture with subsequent stage progression in EPICA cohort. Fig. S11. Analysis strategy for identification of CD8+ subsets characterized by differential expression of TCF-1, PD-1 and TIM-3 in EPICA melanoma lesions by multiple immunofluorescence (mIF). Fig. S12. Correlation analysis in EPICA and TCGA MM cohorts of genes in the HLA Class I APM pathway and clinical significance of the HLA Class I APM signature. Fig. S13. Expression of HLA Class I antigens on tumor cells in representative lesions belonging to the DEM, LOW, INT and CIMP classes of the EPICA cohort. Fig. S14. Expression of melanoma differentiation signatures in EPICA methylation subsets. Fig. S15. Guadecitabine treatment promotes melanoma de-differentiation. Fig. S16. Guadecitabine promotes de-differentiation of the differentiated melanoma clone 2_59. Fig. S17. Guadecitabine treatment of differentiated melan [file 13046_2025_3474_MOESM2_ESM.pdf]

Supplemental Figure S1

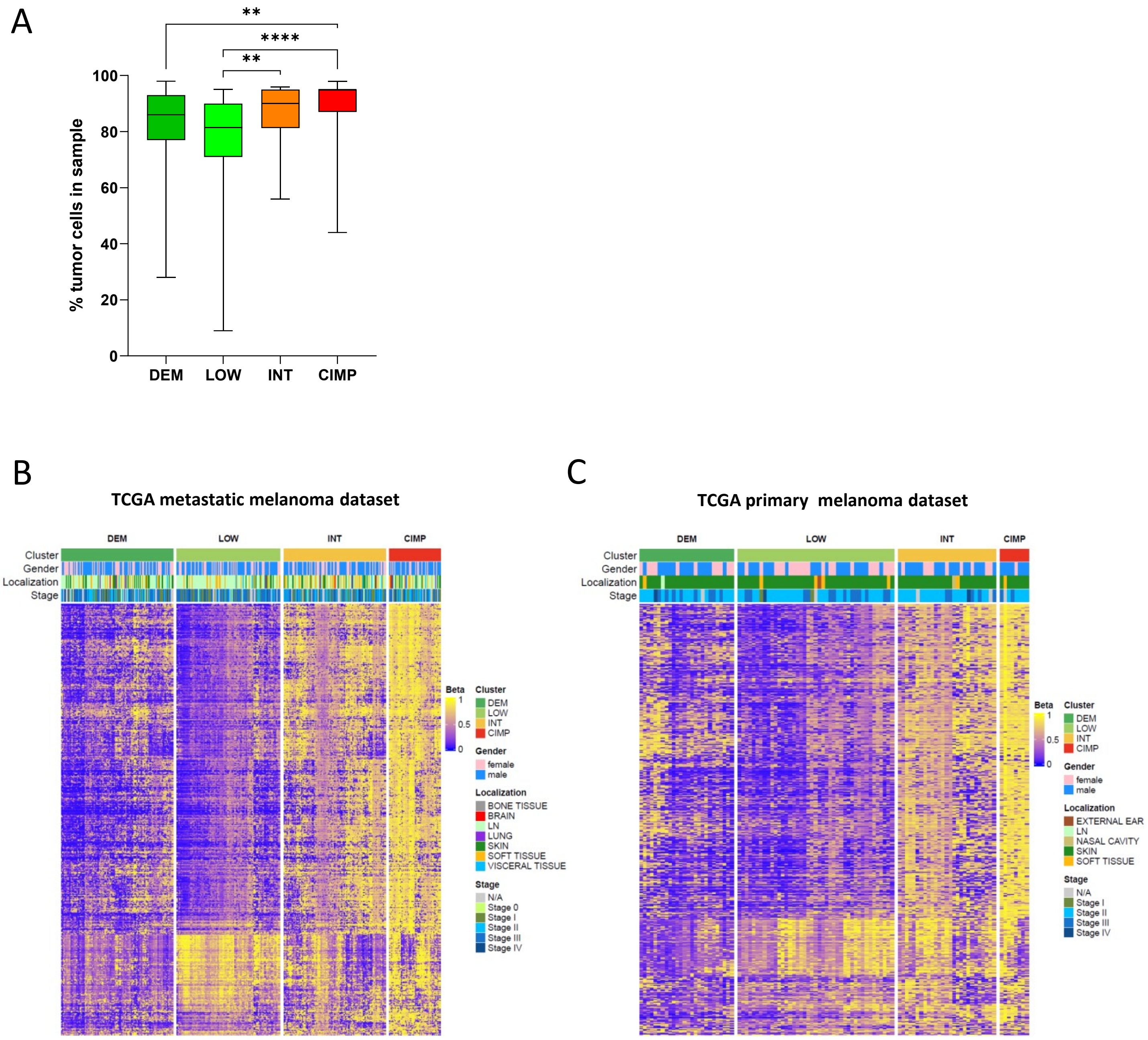

Supplemental Figure S1. **Tumor purity in EPICA methylation subsets and validation of methylation classes in Firehose Legacy TCGA primary and metastatic melanoma cohorts. A** Tumor purity in the 191 EPICA lesions classified for the methylation subset. **B** Unsupervised clustering of n=368 TCGA metastatic SKCM based on 3515 most variable CpG probes. **C** Unsupervised clustering of n=104 primary melanomas cohort from TCGA based on 5605 most variable CpG probes. Statistical analysis in A by Kruskal Wallis test followed by Dunn’s multiple comparison test.

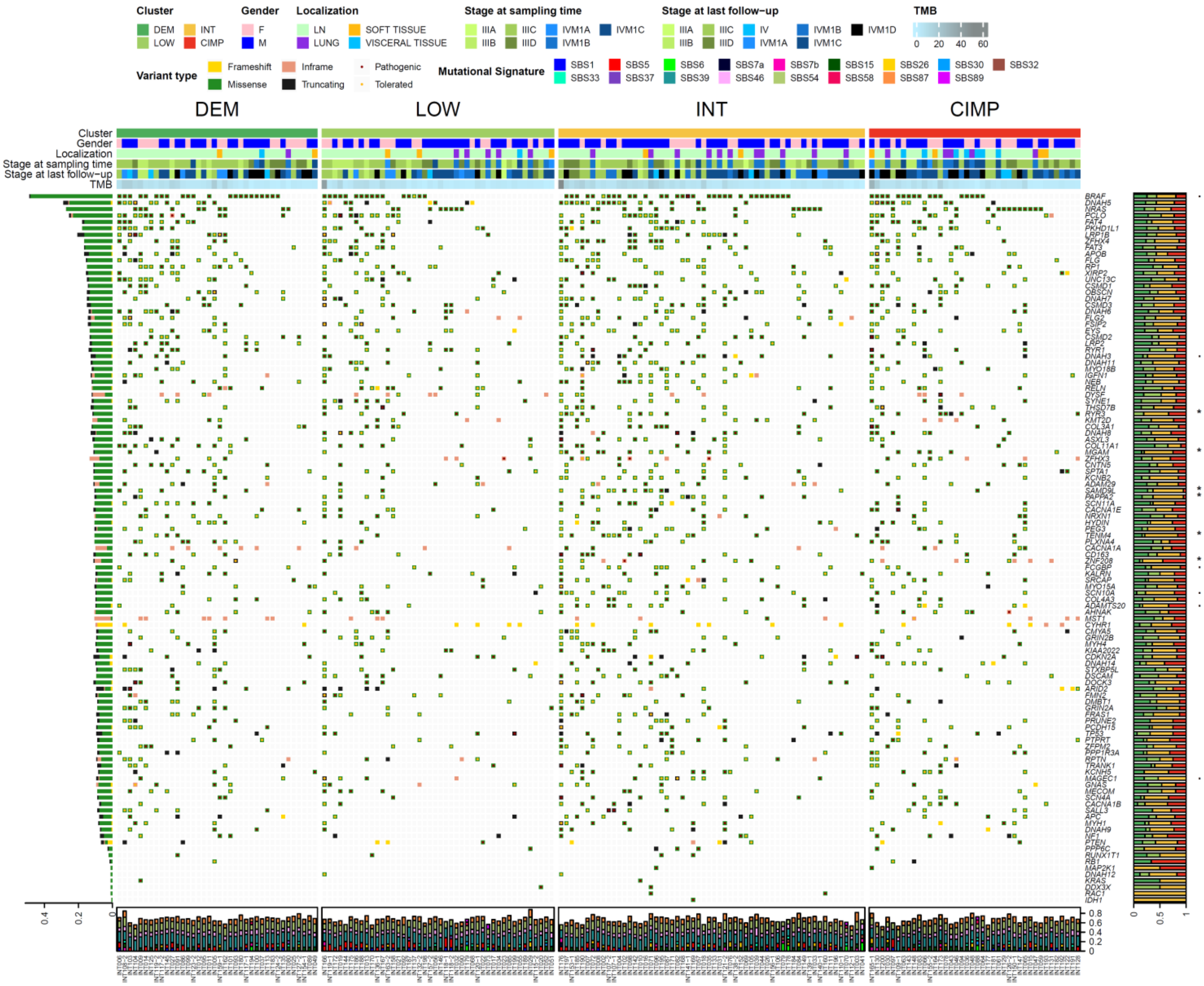

Supplemental Figure S2. **Oncoplot displaying common somatic nonsynonymous alterations in the Stage III/IV EPICA MM cohort, divided by methylation groups**. Rows and columns represent genes and tumor lesions, respectively. Tumor Mutation Burden (TMB) and clinical features for each lesion are shown as tracks at the top. Frequencies of COSMIC mutational signatures are indicated as a bar plot at the bottom. The proportion of alterations among methylation groups for each gene is shown, with statistical significance indicated (p-value of Pearson's chi-squared test statistic: *: p < 0.05, .: p < 0.1).

Supplemental Figure S3

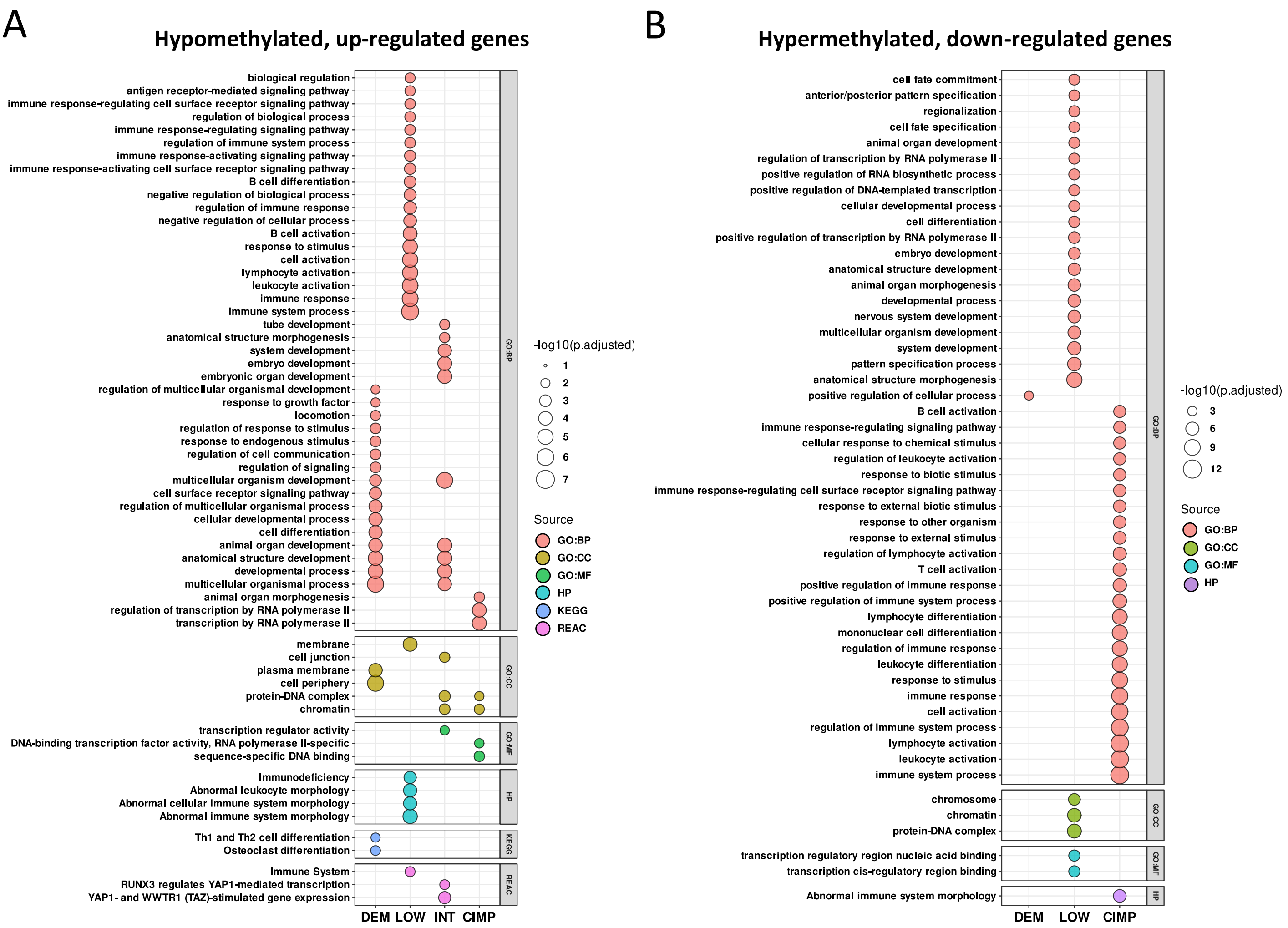

Supplemental Figure S3. Promoter methylation vs. gene expression analysis. A,B Over-representation analysis for association of promoter methylation vs. gene expression for selected gene sets. Dot plots showing the most statistically significantly enriched terms from over-representation analysis (ORA) of up-regulated genes whose promoters are hypomethylated (**A**) and down-regulated genes whose promoters are hypermethylated (**B**), comparing each methylation class vs all the others in EPICA cohort. Dots size represent the adjusted p-values and color codes represent collection of gene sets (GO:BP = Gene Ontology Biological Process; GO:CC = Gene Ontology Cellular Component; GO:MF = Gene Ontology Molecular Function; HP = The Human Phenotype Ontology; KEGG = Kyoto Encyclopedia of Genes and Genomes pathways; REAC = Reactome Pathway.

Supplemental Figure S4

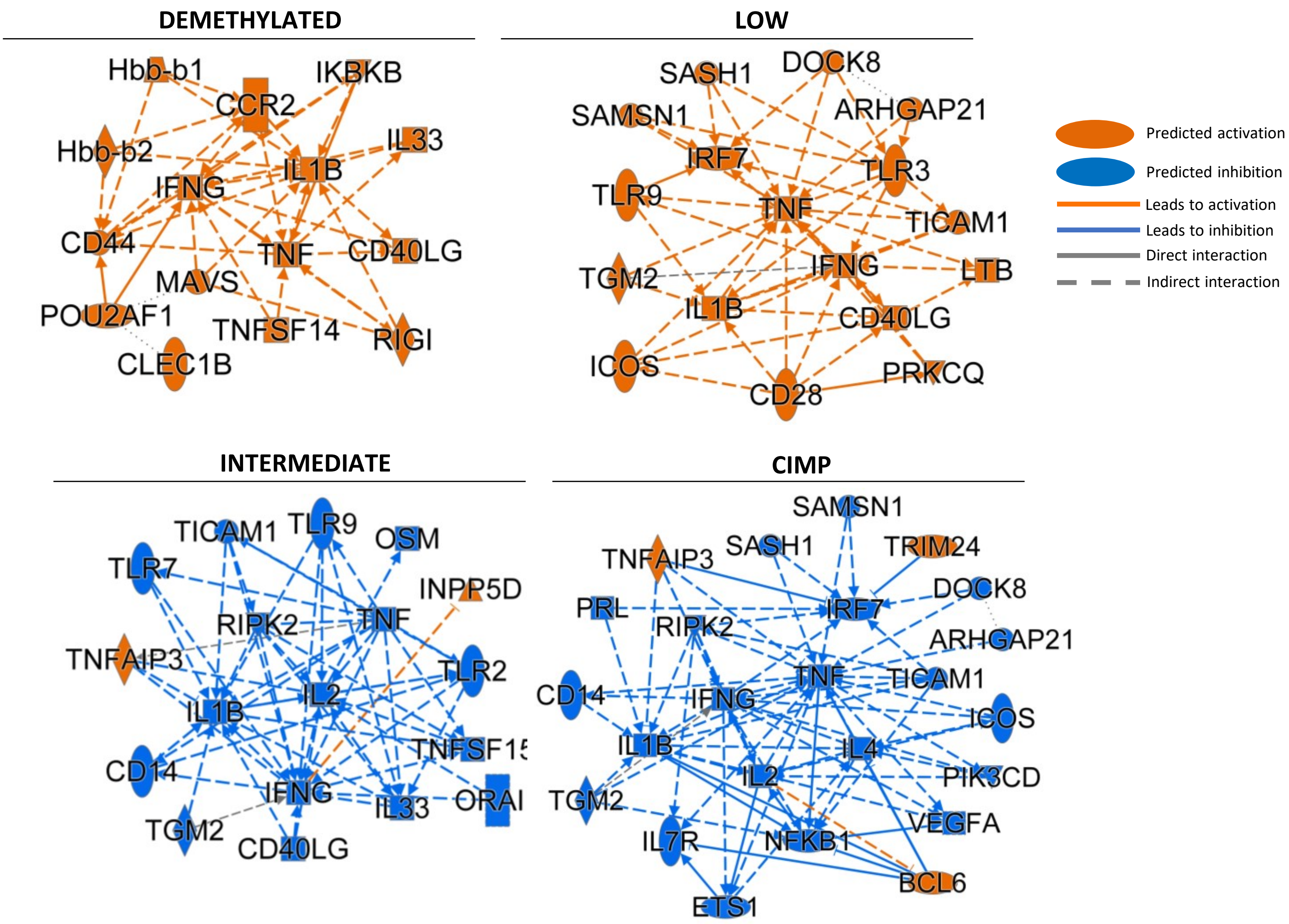

Supplemental Figure S4. IPA core analysis for differentially expressed genes in the DEM, LOW, INT and CIMP EPICA classes. Graphical summary showing the most significant master molecules, their connections and relationships emerging from IPA core analysis for differentially expressed genes in the EPICA methylation classes. The orange and blue nodes mean “predicted activation” or “predicted inhibition”, respectively, of the indicated molecules; orange and blue arrows mean “leads to activation” or “leads to inhibition”, respectively; continuous and dashed lines mean direct or indirect interaction between main nodes, respectively; dotted line means inferred relationship between main nodes.

Supplemental Figure S5

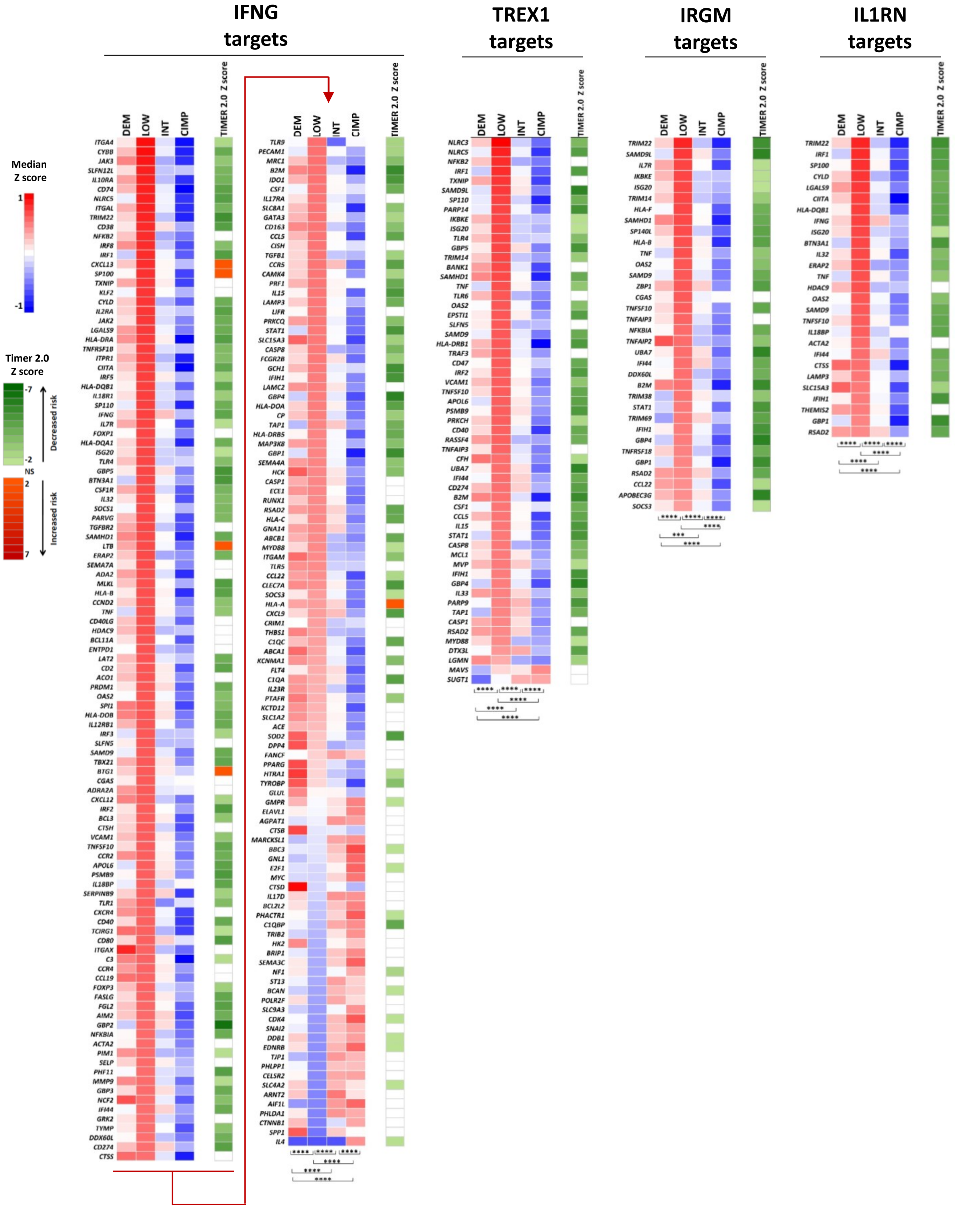

Supplemental Figure S5. Expression and prognostic significance of the IFNG, TREX1, IRGM, and IL1RN target genes in EPICA methylation classes. Heatmaps of median z score expression of IFNG, TREX1, IRGM, and IL1RN target genes in EPICA methylation classes. The column entitled «Timer 2.0 z score» shows the prognostic significance (green: decreased risk; red: increased risk) of each gene as estimated by the Timer 2.0 clinical outcome module in the TCGA MM cohort. Statistical analysis by Kruskal Wallis test followed by Dunn's multiple comparison test. *: $p<0.05$; **: $p<0.01$, ***: $p<0.001$; ****: $p<0.0001$.

Supplemental Figure S6

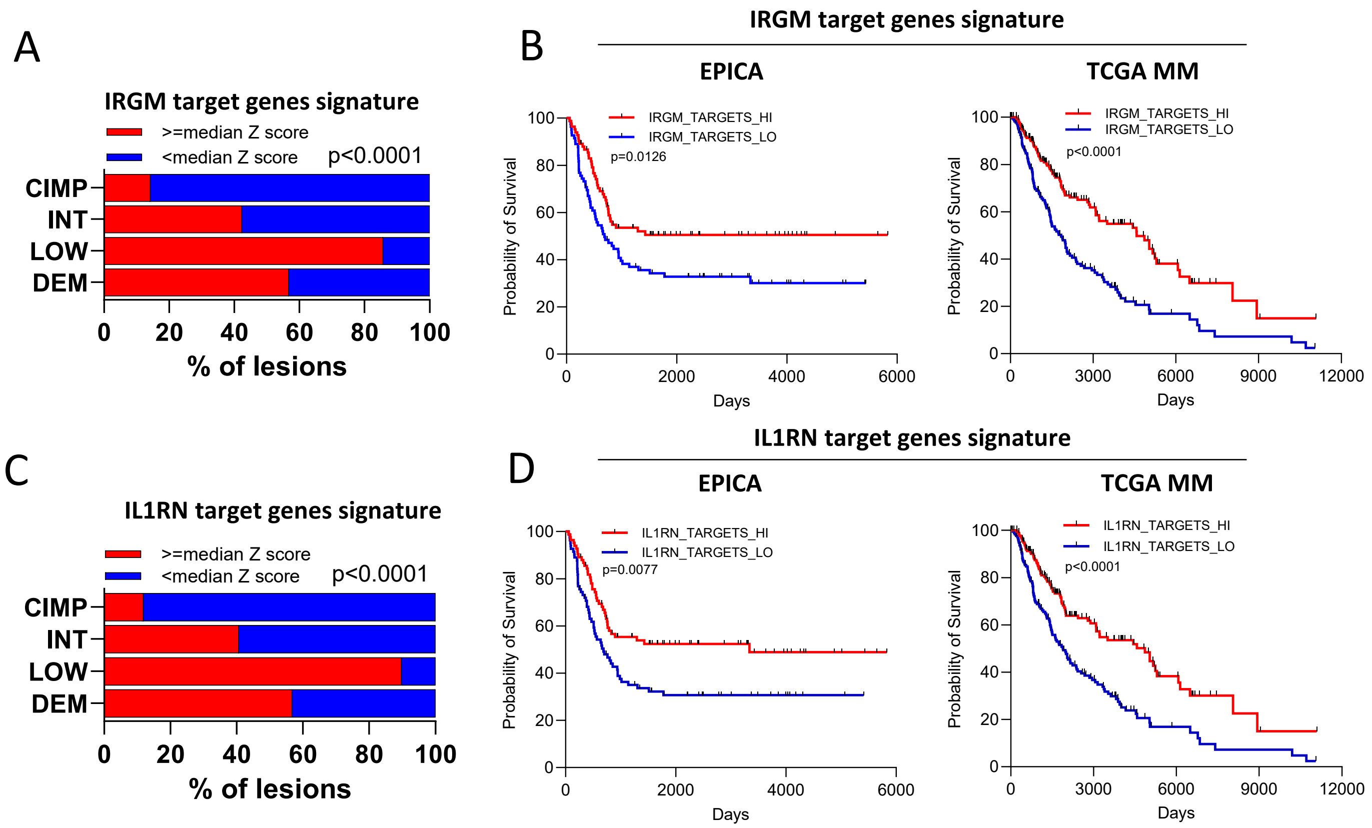

Supplemental Figure S6. Top master molecules activated in CIMP MM classes are negative regulators of target genes with prognostic significance. A,C Stacked bar plots showing, for each methylation cluster in the EPICA cohort, the percentage of samples with expression of the IRGM (**A**) or IL1RN (**C**) target genes above or below the median z score value of each signature. **B**,**D** Kaplan-Meier survival curves of patients in the EPICA cohort (left plot) or TCGA MM cohort (right plot) according to median z score expression of IRGM (**B**) or IL1RN (**D**) target gene signatures**.** In **B**,**D** patients in both cohorts were grouped according to median UR target gene expression above ("HI") or below ("LO") the median z score value of the signature. Statistical analysis in **A**, **C** by chi square; in **B**, **D** by log rank test.

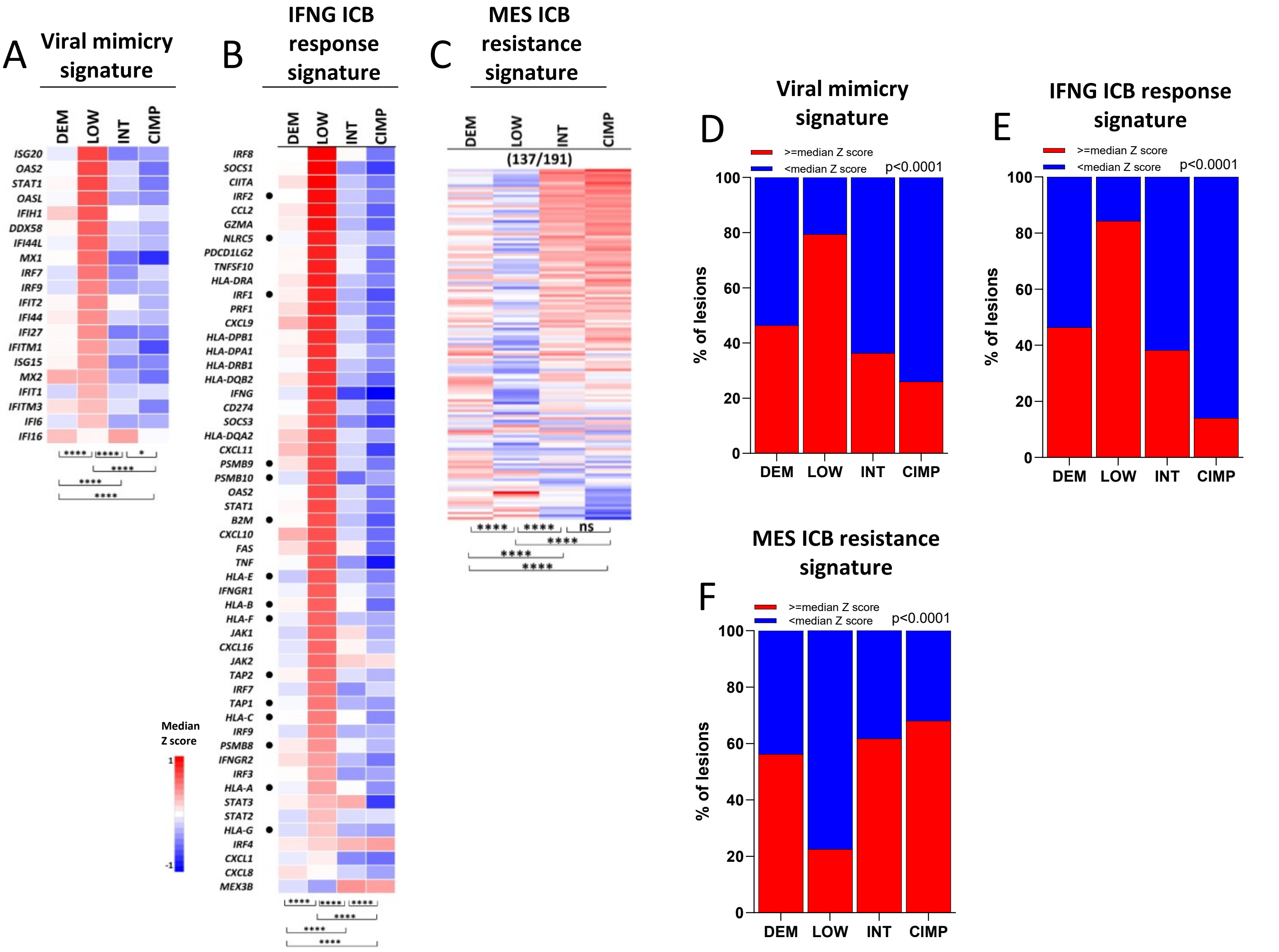

Supplemental Figure S7. Expression of ICB predictive signatures in methylation-defined classes of the TCGA MM cohort. A,B,C Heatmaps of median z score expression of genes in the viral mimicry (**A**), IFN ICB response (**B**) and MES resistance (**C**) signatures in methylation defined classes of the TCGA MM cohort. Genes identified by dots in panel **B** represent a core HLA Class I APM signature. **D**,**E**,**F** Stacked bar plots showing for each methylation cluster in the TCGA MM cohort the percentage of samples with expression above or below the median z score value of each signature for the viral mimicry (**D**), the IFNG ICB response (**E**), and the MES resistance (**F**) signatures. Statistical analysis in **A**-**C** by Kruskal Wallis test followed by Dunn's multiple comparison test; in **D**-**F** by Chi-square. *: p<0.05; **: p<0.01, ***: p<0.001; ****: p<0.0001.

Supplemental Figure S8

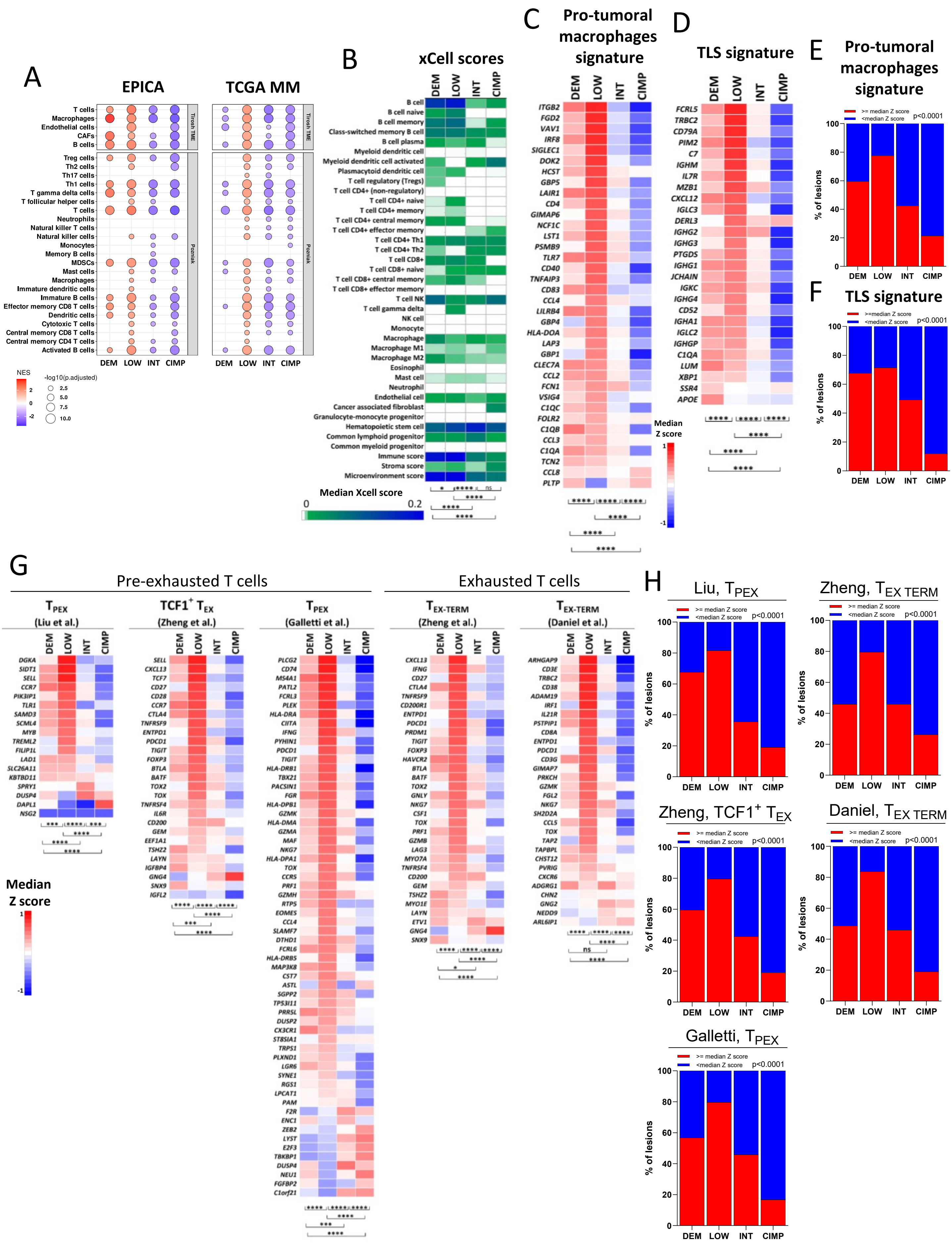

Supplemental Figure S8. Assessment of immune-related gene signatures in the EPICA methylation-defined clusters. **A** Dot plot of normalized enrichment scores (NES) of two melanoma microenvironment signatures [45, 46] in the four methylation subsets of the EPICA and TCGA metastatic melanoma cohorts. Dot size represents the adjusted p-values and scale colors represent the NES. **B** Heatmap of median xCell algorithm scores in DEM, LOW INT and CIMP classes. **C,D** Heatmaps of expression median z scores in the four methylation subsets computed for genes belonging to the pro-tumoral macrophage signature [48] (**C**) and TLS gene signature [49] (**D**). **E,F** Stacked bar plots showing for each methylation cluster the percentage of samples with expression of the pro-tumoral macrophages (**E**) or TLS (**F**) signatures above or below the median z score value of each signature. **G** Heatmap of expression median z scores in the four methylation subsets computed for genes belonging to three T_{PEX} signatures [50-52] and to two T_{EX} signatures [52,53]. **H** Stacked bar plots showing for each methylation cluster the percentage of samples with expression of the T_{PEX} and T_{EX} gene signatures above or below the median z score value of each signature. Statistical analysis by Kruskall Wallis test followed by Dunn's multiple comparison test in **B**,**C**,**D** and **G** and by Chi-square in **E**,**F**,**H**.*: p<0.05; **: p<0.01, ***: p<0.001; ****: p<0.0001

Supplemental Figure S9

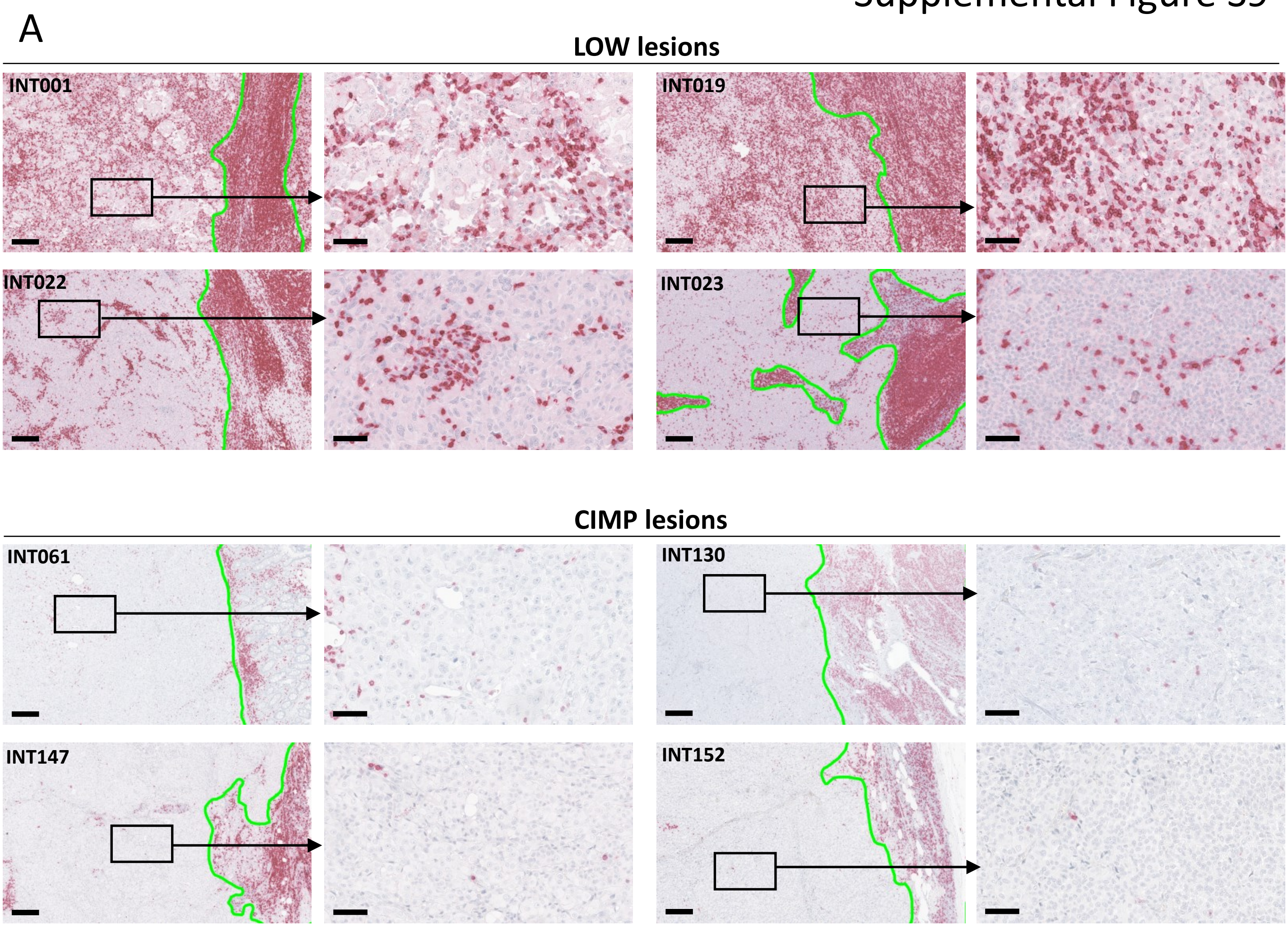

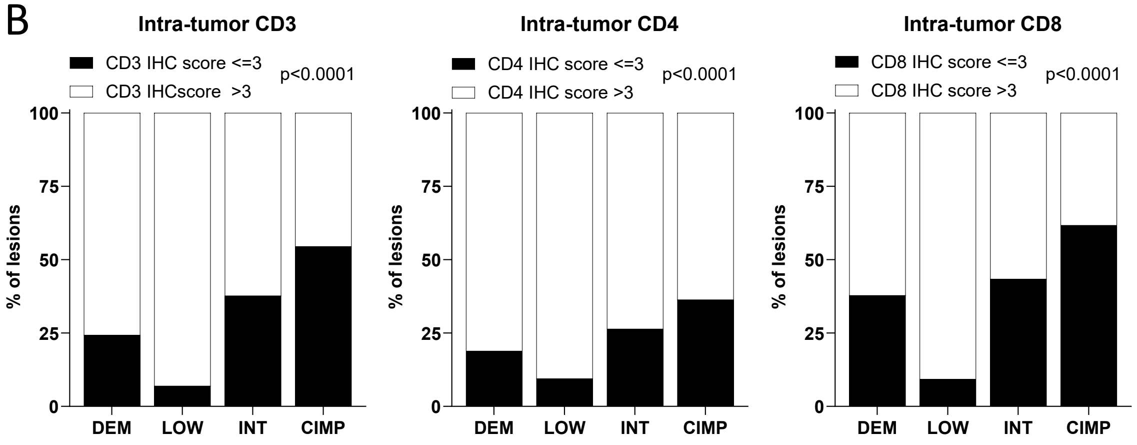

Supplemental Figure S9. LOW lesions are enriched for intra-tumor T cells compared to CIMP lesions. **A** representative images of four LOW lesions (TOP panels) and of four CIMP lesions (bottom panels) stained for CD3. For each lesion a higher magnification panel shows an intra-tumor area. The green line identifies the intra-tumor (left side of each image) and the extra-tumor (right side of each image) areas of each lesion. Scalebar: 200 μ (lower magnification image) and 50 μ (higher magnification image). **B** Fraction of lesions in each methylation class having an intra-tumor CD3, CD4 and CD8 IHC score <=3 or >3. Statistical analysis by Chi-square.

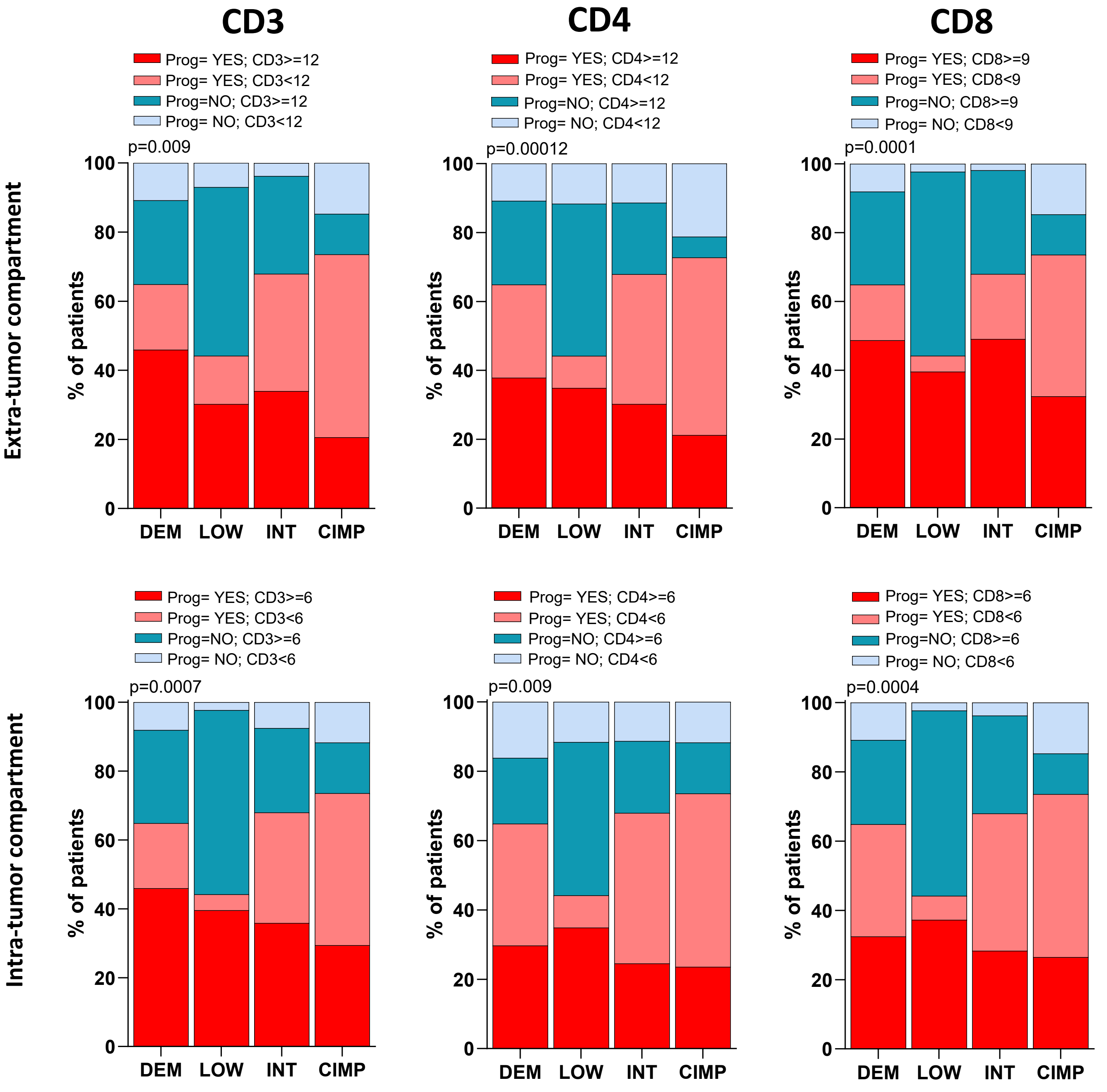

Supplemental Figure S10. Association of methylation classes and tumor immune contexture with subsequent stage progression in EPICA cohort. Stacked bar plots of the percentages of EPICA patients, in each methylation class, grouped according to level of expression (above or below the median IHC score) of CD3, CD4 and CD8 in extra- or intra-tumor compartments in the initial investigated lesion and according to subsequent AJCC stage progression (Prog =YES, light red or dark red bars) or to lack of subsequent stage progression (Prog=NO, light blue or dark blue bars). Statistical analysis by Chi-square.

Supplemental Figure S11

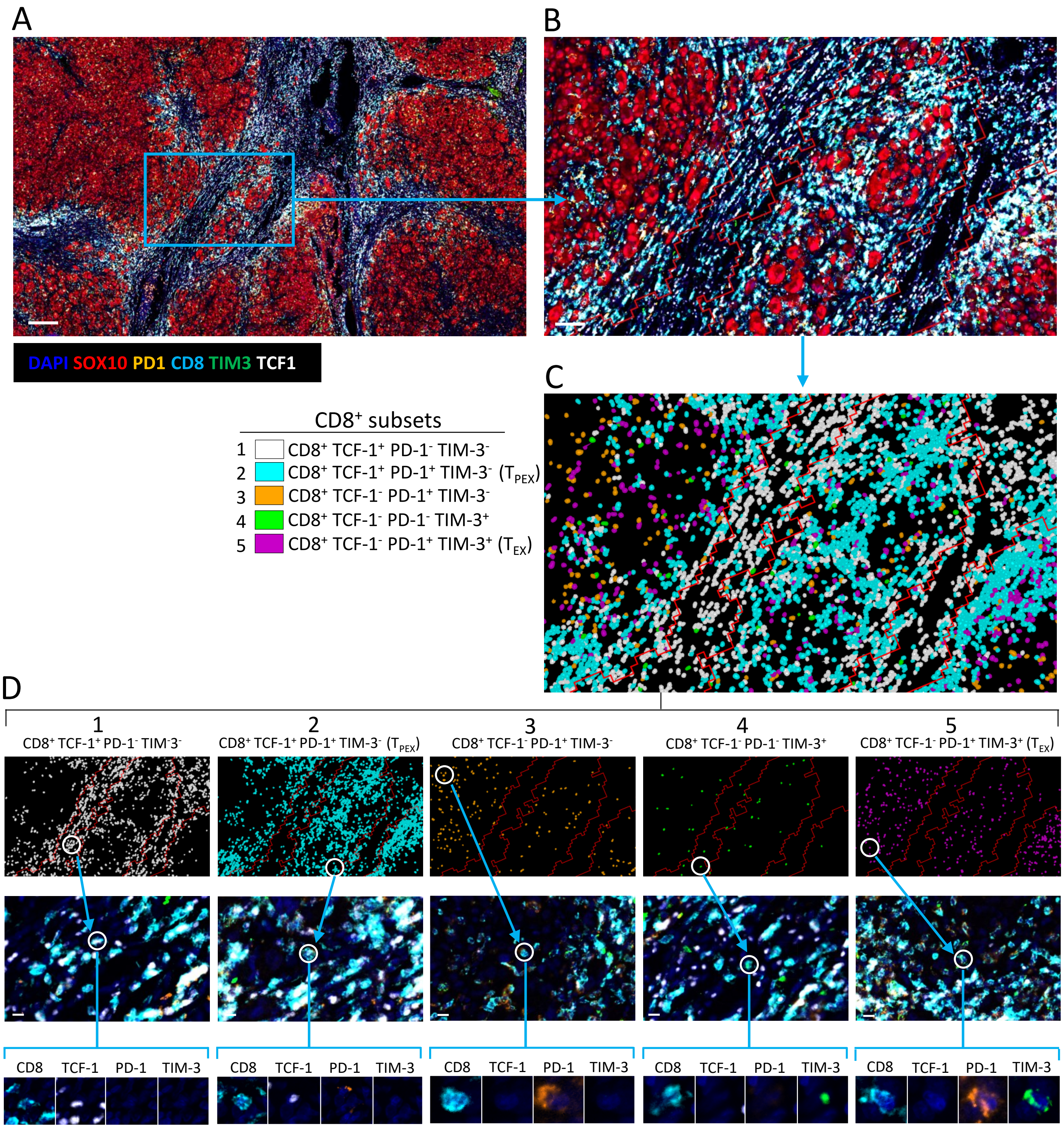

Supplemental Figure S11. Analysis strategy for identification of $CD8^+$ subsets characterized by differential expression of TCF-1, PD-1 and TIM-3 in EPICA melanoma lesions by multiple immunofluorescence (mIF). A mIF image of a metastatic melanoma lesion (scale bar:200μ). **B** A higher magnification area (scale bar: 100 μ) of the lesion in **A**. Red lines: tissue segmentation to discriminate tumor from stroma areas. **C** Identity and position in the same area as in panel b, of $CD8^+$ cells expressing the 5 indicated phenotypes. **D** Upper panels: position of each of the identified $CD8^+$ phenotypes in the same area as in **C**; middle panels: original mIF images centered on a higher magnification area containing representative examples of each $CD8^+$ phenotype (scale bar: 10 μ); bottom panels: single channel mIF images showing expression of CD8, TCF-1, PD-1, TIM-3 in a single cell representative of each of the five identified subsets. Visualization of tumor cells was omitted in panel **C** and **D**.

Supplemental Figure S12

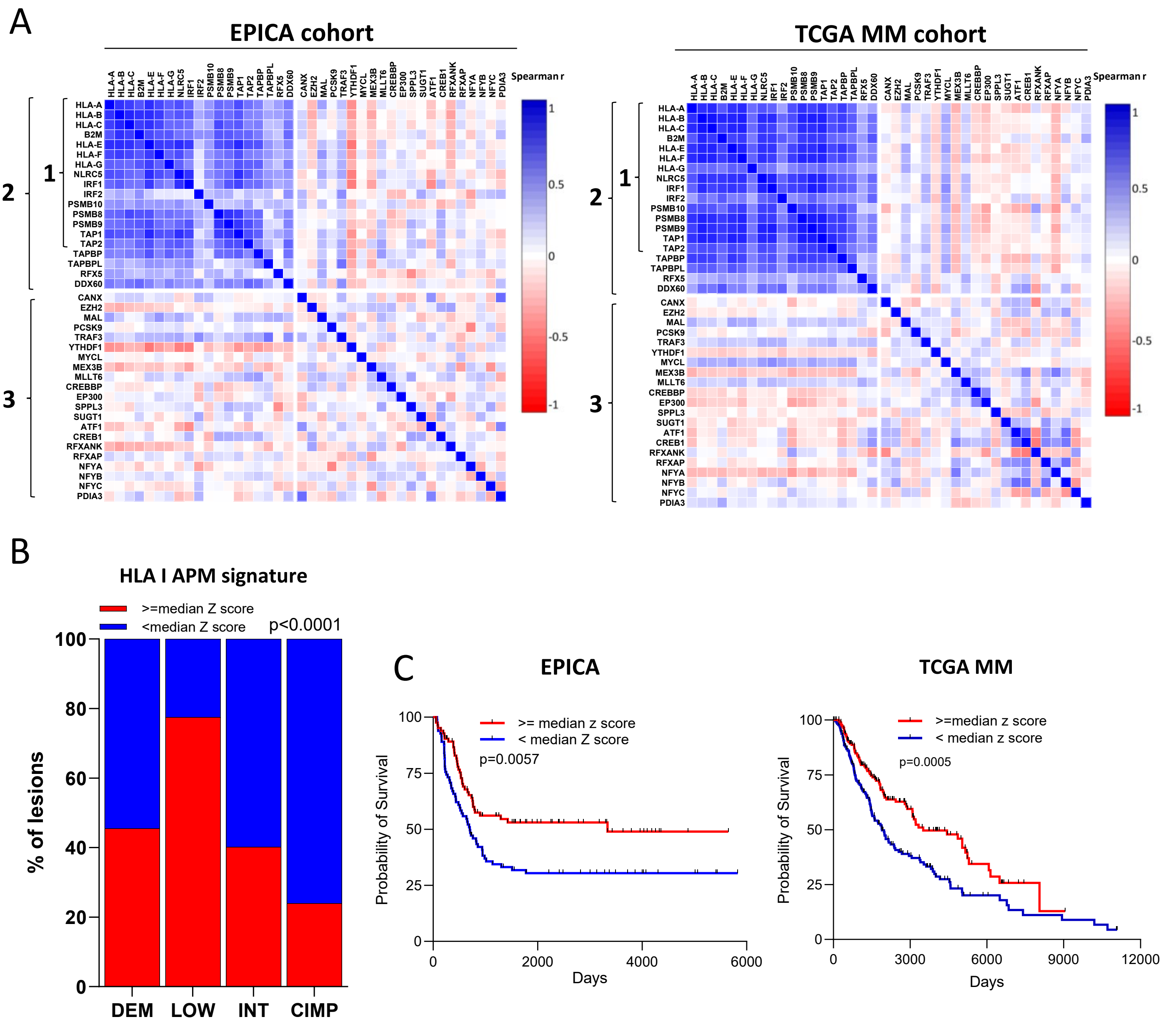

Supplemental Figure S12. **Correlation analysis in EPICA and TCGA MM cohorts of genes in the HLA Class I APM pathway and clinical significance of the HLA Class I APM signature**. **A** Spearman correlation analysis in EPICA and TCGA MM cohorts of the expression levels of genes in the core HLA class I APM signature (labelled “1”), in the extended HLA Class I APM signature (labelled “2”) and of genes involved in positive and negative regulation of HLA Class I expression (labelled “3”). **B** Stacked bar plots showing for each methylation cluster in the EPICA cohort the percentage of samples with expression above or below the median z score value of the HLA Class I APM signature. **C** Kaplan-Meier survival curves of patients in the EPICA cohort and in the TCGA MM cohort according to median z score value of the extended HLA Class I APM signature 2. Statistical analysis in **A** by spearman correlation, in **B** by Chi-square, in **C** by log-rank test.

Supplemental Figure S13

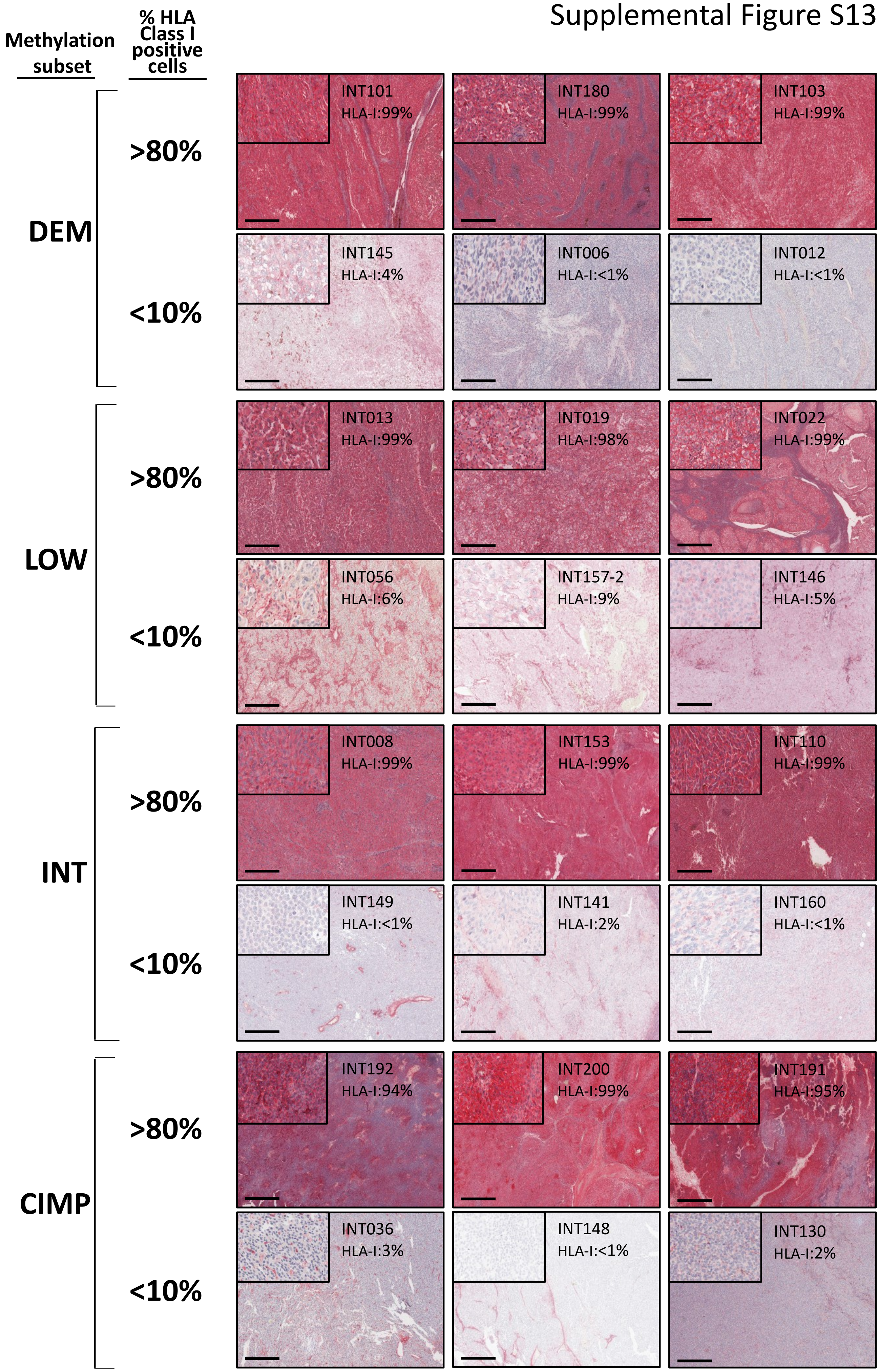

Supplemental Figure S13. Expression of HLA Class I antigens on tumor cells in representative lesions belonging to the DEM, LOW, INT and CIMP classes of the EPICA cohort. Six representative lesions from each methylation class, stained in IHC for HLA Class I antigens and evaluated by quantitative digital pathology analysis are shown. For each methylation class three lesions with high expression of HLA Class I on tumor cells and three lesions with strong HLA Class I downmodulation are shown. For each lesion the inset shows a higher magnification of a tumor area. Scale bar: 500μ.

Supplemental Figure S14

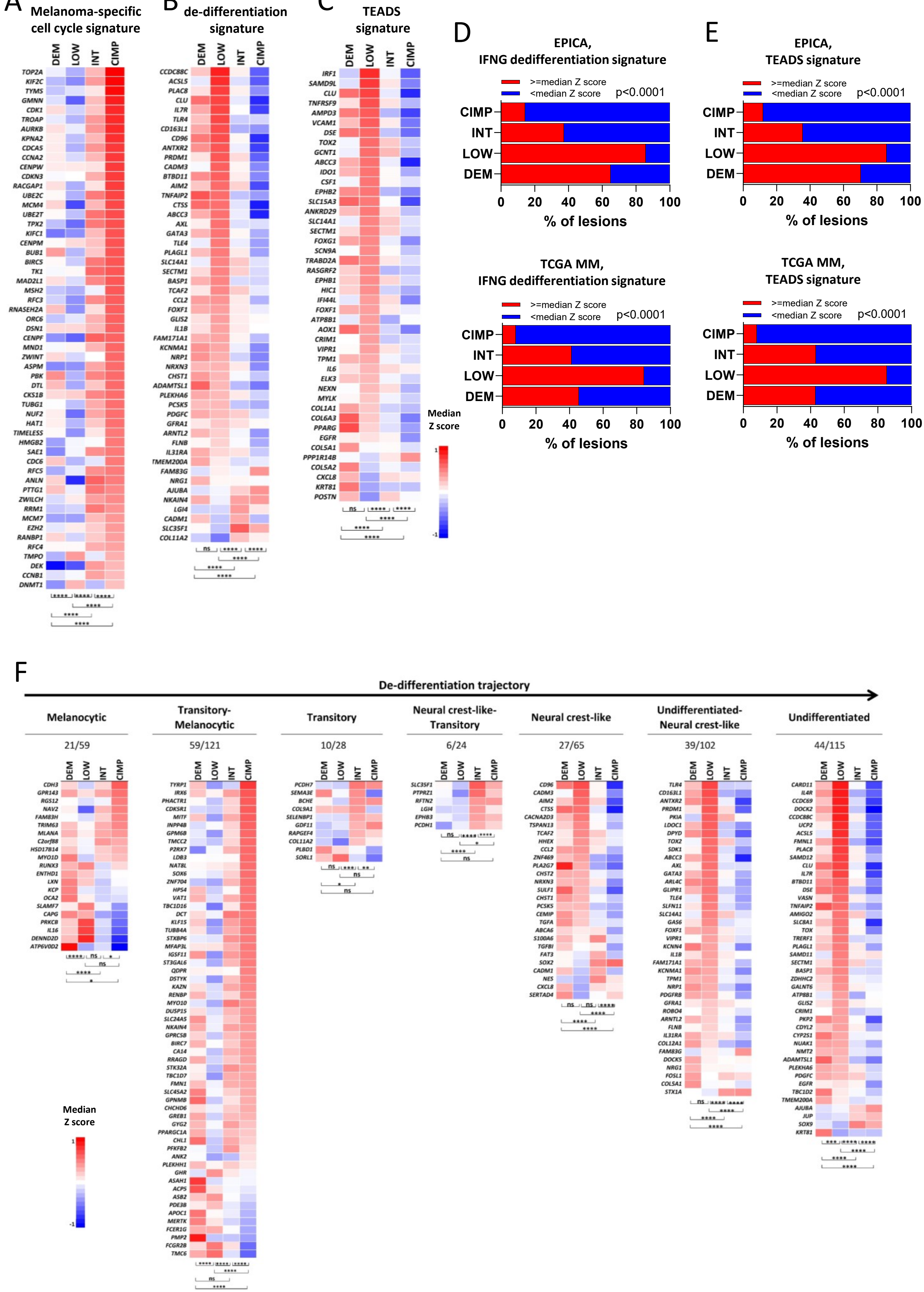

Supplemental Figure S14. Expression of melanoma differentiation signatures in EPICA methylation subsets. A,B,C Heatmaps of median z score expression for genes in (**A**) of the melanoma-specific cell cycle signature [45], in (**B**) of 50/111 genes in the IFNG de-differentiation signature [57] and in (**C**) of 44/107 genes in the TEADS signature [58] that discriminate the methylation defined classes of the EPICA cohort. **D,E** Stacked bar plots showing for each methylation cluster in the EPICA cohort (Top graph) and TCGA metastatic melanoma cohort (bottom graph) the percentage of samples with expression of the IFNG de-differentiation (**D**) and TEADS signatures (**E**) above or below the median z score value of the signature. **F** Heatmaps showing median z score values from RNA-seq profiling for selected genes in the seven sub-signatures describing melanoma differentiation according to Tsoi et al. [59]. Number of genes that discriminate methylation groups/total number of genes in each subsignature is shown below the sub-signature name. Statistical analysis in **A**, **B**, **C**, **F**: Kruskal Wallis test followed by Dunn's multiple comparison test; in **D**,**E** by Chi-square. *: $p<0.05$, **: $p<0.01$; ***: $p<0.001$, ****: $p<0.0001$.

Supplemental Figure S15

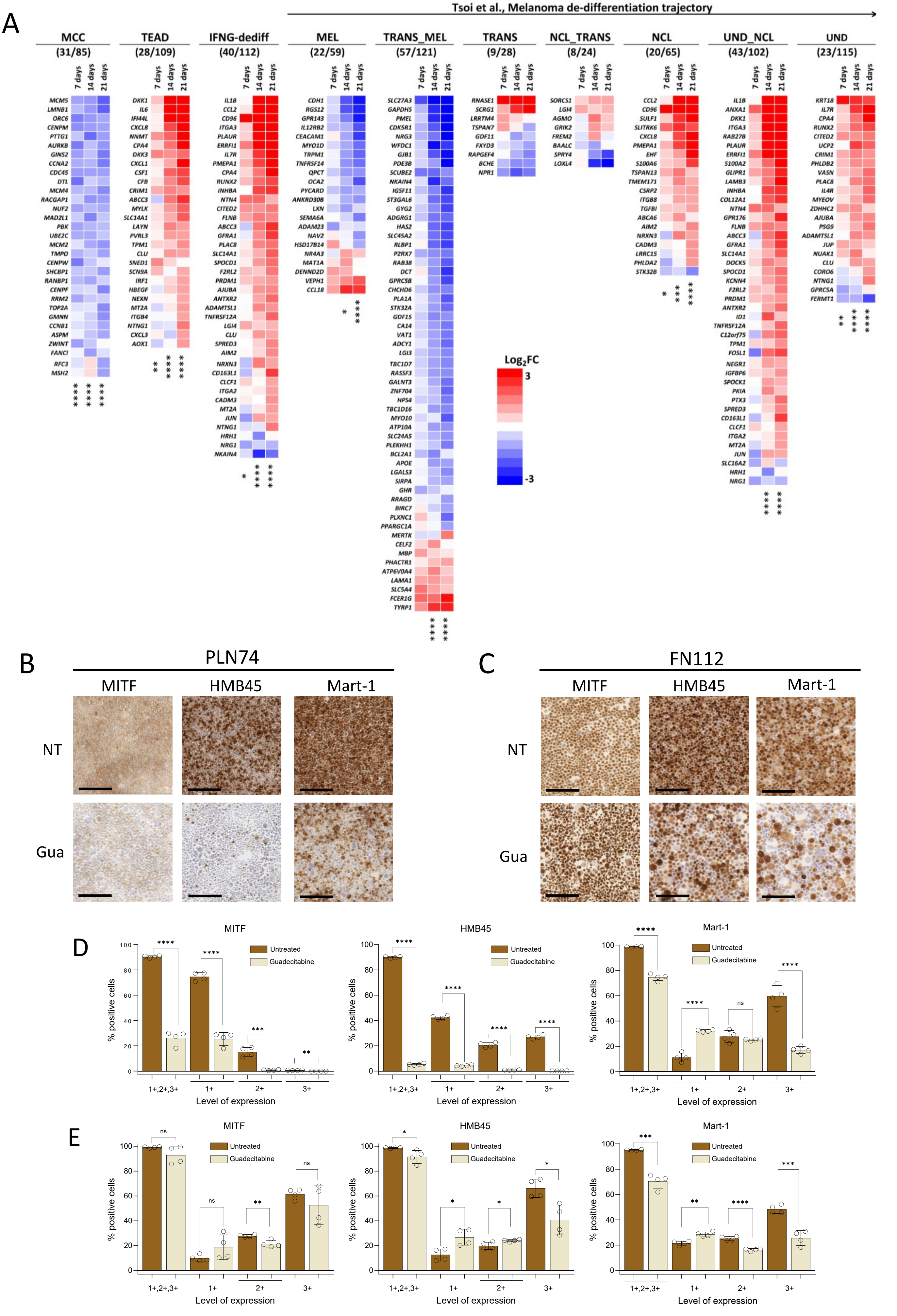

Supplemental Figure S15. Guadecitabine treatment promotes melanoma de-differentiation. A Heatmaps showing modulation of genes in the MCC [45], TEADS [58], IFNG-dediff [57] and Tsoi et al. [59] melanoma differentiation signatures in cell line PLN74 at day 7, 14 and 21 of treatment. For each gene signature only genes showing a $Log_2FC>|1|$ are shown. The number of these genes/the total number of genes in each signature is shown under the signature name. Statistical analysis by Mann Whitney test. **B,C** Modulation of MITF, HMB45 and Mart-1 protein expression in two differentiated melanoma cell lines by guadecitabine. Images from cytospins of cell lines PLN74 (**B**) and FN112 (**C**) cultured or not with guadecitabine for 21 days and then stained with mAbs to MITF, HMB45 (PMEL/GP100) and MART-1 melanoma markers. Scale-bar = 200µm. **D,E** Quantitative digital pathology analysis by QuPath software of MITF, HMB45 and Mart-1 expression in cell lines PLN74 (**D**) and FN112 (**E**) cultured or not with guadecitabine for 21 days. Results expressed as % positive cells at any level of expression (1+,2+,3+) or at levels 1+, 2+ or 3+. Statistical analysis in **D**,**E** by Student T test. *: $p<0.05$; **:$p<0.01$, ***:$p<0.001$; ****$p<0.0001$.

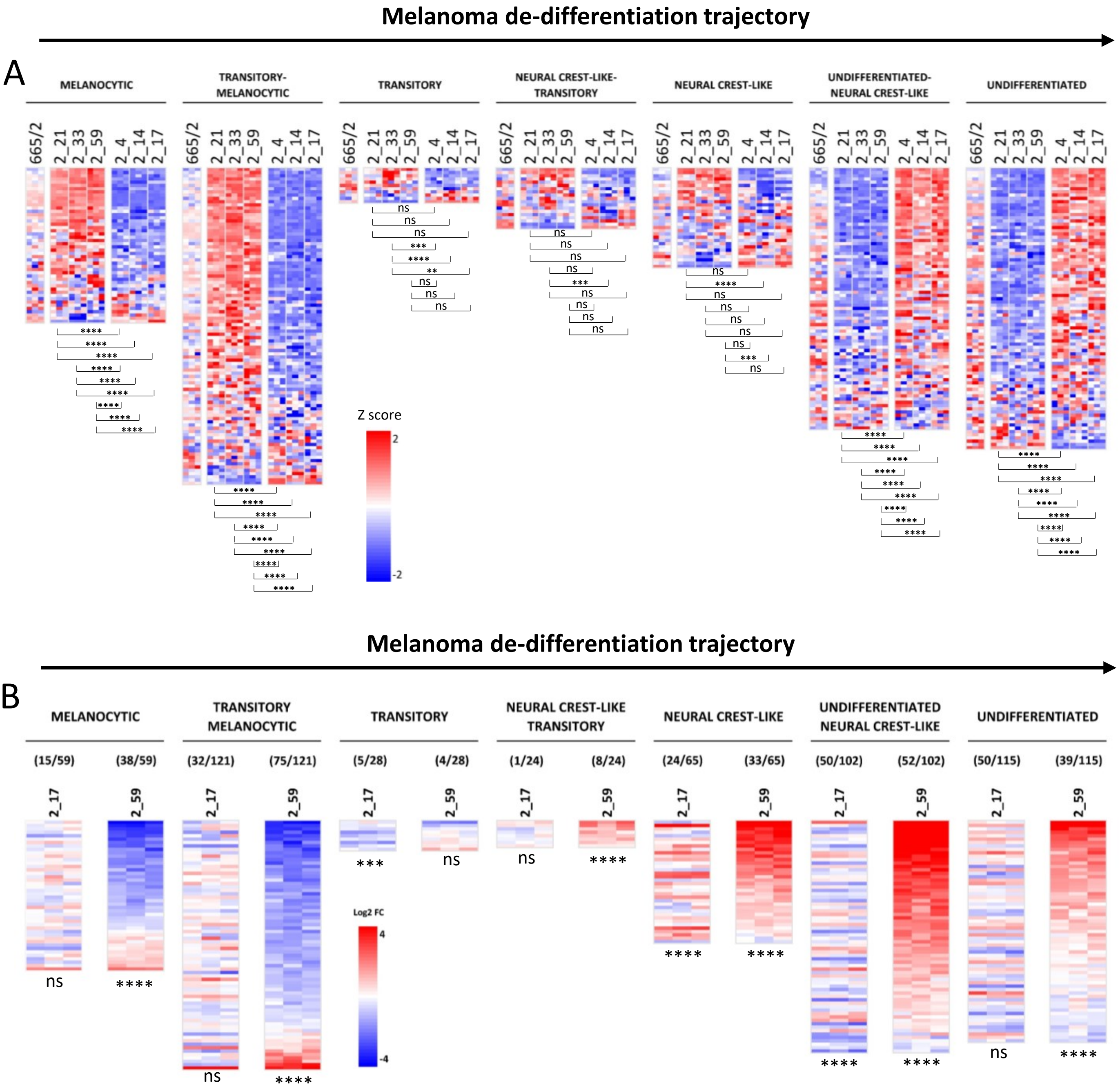

Supplemental Figure S16. Guadecitabine promotes de-differentiation of the differentiated melanoma clone 2_59. **A** Heatmaps showing expression of genes in the seven melanoma differentiation sub-signatures (Tsoi et al.[59]) in the parental line 665/2 and in six clones (2_21_2_33, 2_59, 2_4, 2_14, 2_17) generated from the parental line 665/2. Two groups of clones can be identified. For each sub-signature, only genes discriminating clones 2_21, 2_33 and 2_59 from clones 2_4, 2_14 and 2-17 are shown. **B** Modulation of genes in the seven melanoma differentiation sub-signatures (Tsoi et al.[59]) by treatment with guadecitabine in melanoma clones 2_17 and 2_59. For each sub-signature, only genes showing significant modulation by guadecitabine in each of the two clones are shown. The number of these genes/the total number of genes in each sub-signature is shown under the signature name. Statistical analysis in **A**, **B** by two way ANOVA followed by Tukey's multiple comparison test. *: p<0.05; **:p<0.01; ***:p<0.001; ****:p<0.0001.

Supplemental Figure S17

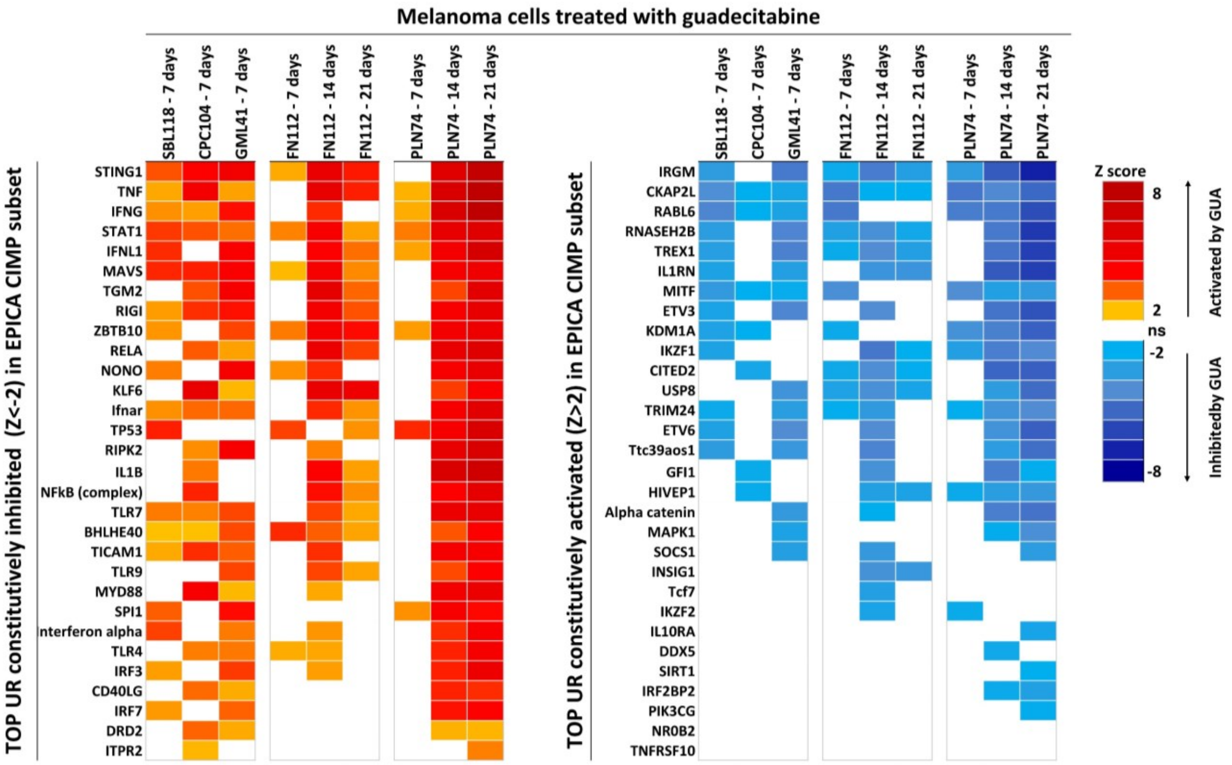

Supplemental Figure S17. Guadecitabine treatment of differentiated melanoma lines reverses the activation state of UR identified in CIMP lesions. TOP UR constitutively inhibited in CIMP tumors (left) or constitutively activated in CIMP tumors (right) were selected and tested by IPA UR analysis for change in their activation state in melanoma cell lines treated at 7, 14 or 21 days with guadecitabine. The heatmaps show the significantly activated (orange to red) and significantly inhibited (light blue to blue) UR upon guadecitabine treatment based on z score values.
